# FMNL formins boost lamellipodial force generation

**DOI:** 10.1038/ncomms14832

**Published:** 2017-03-22

**Authors:** Frieda Kage, Moritz Winterhoff, Vanessa Dimchev, Jan Mueller, Tobias Thalheim, Anika Freise, Stefan Brühmann, Jana Kollasser, Jennifer Block, Georgi Dimchev, Matthias Geyer, Hans-Joachim Schnittler, Cord Brakebusch, Theresia E. B. Stradal, Marie-France Carlier, Michael Sixt, Josef Käs, Jan Faix, Klemens Rottner

**Affiliations:** 1 Division of Molecular Cell Biology, Zoological Institute, Technische Universität Braunschweig, Spielmannstrasse 7, 38106 Braunschweig, Germany; 2 Molecular Cell Biology Group, Helmholtz Centre for Infection Research, Inhoffenstrasse 7, 38124 Braunschweig, Germany; 3 Institute for Biophysical Chemistry, Hannover Medical School, Carl-Neuberg-Strasse 1, 30625 Hannover, Germany; 4 Institute of Science and Technology Austria, Am Campus 1, 3400 Klosterneuburg, Austria; 5 Soft Matter Physics Group, Institut für experimentelle Physik I, Leipzig University, Linnéstraße 5, 04103 Leipzig, Germany; 6 Biomedical Institute, BRIC, University of Copenhagen, DK-2200 Copenhagen, Denmark; 7 Department of Cell Biology, Helmholtz Centre for Infection Research, Inhoffenstrasse 7, 38124 Braunschweig, Germany; 8 Institute of Innate Immunity, Department of Structural Immunology, University of Bonn, Sigmund-Freud-Strasse 25, 53127 Bonn, Germany; 9 Institute of Anatomy and Vascular Biology, Westfälische Wilhelms-Universität Münster, Vesaliusweg 2-4, 48149 Münster, Germany; 10 Cytoskeleton Dynamics and Motility Group, Laboratoire d'Enzymologie et Biochimie Structurales, Centre de Recherche de Gif, CNRS, Gif-sur-Yvette 91198, France

## Abstract

Migration frequently involves Rac-mediated protrusion of lamellipodia, formed by Arp2/3 complex-dependent branching thought to be crucial for force generation and stability of these networks. The formins FMNL2 and FMNL3 are Cdc42 effectors targeting to the lamellipodium tip and shown here to nucleate and elongate actin filaments with complementary activities *in vitro*. In migrating B16-F1 melanoma cells, both formins contribute to the velocity of lamellipodium protrusion. Loss of FMNL2/3 function in melanoma cells and fibroblasts reduces lamellipodial width, actin filament density and -bundling, without changing patterns of Arp2/3 complex incorporation. Strikingly, in melanoma cells, *FMNL2/3* gene inactivation almost completely abolishes protrusion forces exerted by lamellipodia and modifies their ultrastructural organization. Consistently, CRISPR/Cas-mediated depletion of FMNL2/3 in fibroblasts reduces both migration and capability of cells to move against viscous media. Together, we conclude that force generation in lamellipodia strongly depends on FMNL formin activity, operating in addition to Arp2/3 complex-dependent filament branching.

## Abstract (web summary)

Actin polymerization in lamellipodia of cells is regulated by the Arp2/3 complex and FMNL family formins. Here the authors show that both FMNL2 and FMNL3 contribute to lamellipodium protrusion and structure, and abolishing FMNL2/3 reduces protrusion force generation and migration, without affecting Arp2/3 incorporation.

During migration, cells can form multiple types of protrusions, all of which are employed with high flexibility depending on biochemical and/or mechanical features of their microenvironments^1^,^2^. The best known protrusion types expressed at varying extent in different cell types are sheet-like lamellipodia, finger-like filopodia and membrane blebs, the formation of all of which depends on the actin cytoskeleton.

Lamellipodia are reasonably well understood, thus constituting an excellent model system to examine the relative, mechanistic functions of distinct actin-binding proteins that cooperate in protrusion. It is commonly agreed that Rac drives the generation of lamellipodial actin filament networks through Arp2/3 complex-mediated branching at the interface of polymerizing actin filaments and protruding plasma membrane^3^. Coincident signals including Rac and the phosphoinositide PIP_3_ (phosphatidyl-3,4,5-trisphosphate) stimulate activation of the heteropentameric WAVE complex^4^,^5^, which appears essential for Arp2/3 complex activation in lamellipodia^6^,^7^,^8^. Consistently, functional interference with Arp2/3 complex eliminates lamellipodia formation entirely^9^,^10^,^11^, but whether or not Arp2/3 complex-dependent branching of actin filaments is sufficient for the generation of lamellipodial actin networks, and how other actin filament assembly factors contribute to this process *in vivo* has mostly remained unclear. For instance, aside from Arp2/3 complex, lamellipodial actin assembly is thought to be promoted by Ena/VASP family proteins or formins^3^,^12^, but the exact relative contributions of these protein families to protrusion are unknown.

Formin family proteins can modulate actin filament dynamics by various means, including actin filament bundling or even severing^13^, but the most common activities are nucleation of actin filaments and their processive elongation in a profilin-dependent fashion^14^,^15^. In many cell types, formins are best known for their potential function in driving the assembly of linear actin filaments and bundles in filopodia^16^,^17^,^18^,^19^, although no particular formin is as yet recognized as essential for the formation of these structures^20^,^21^. However, two formin subfamilies have previously been implicated in contributing to lamellipodium protrusion, the Dia subfamily including mDia1 or mDia2 (human DRF3) and the FMNL subfamily including FMNL2. Each of these two formin subfamilies comprises three members, with FMNL1, formerly called FRL1, being restricted in expression to leukocytes and certain epithelial cell lines (see ref. 22 and below). However, the functions exerted by these formins in lamellipodia are not well understood. mDia1 has recently been suggested to produce mother filaments for Arp2/3-dependent branching^23^, but the protein accumulates at the rear cortex^24^ instead of the lamellipodium tip where branching takes place^25^, and fibroblasts derived from mDia1 knockout cells readily form lamellipodia^26^. Likewise, mDia2 has been implicated as a mother filament generator in lamellipodia of B16-F1 melanoma (B16) cells^19^, but its accumulation at lamellipodia tips of these cells appeared as exception rather than rule^16^.

As opposed to this, FMNL2 and -3, two more ubiquitously expressed members of the FMNL subfamily in mammals^27^,^28^ clearly target to lamellipodia, both as expressed, constitutively active versions or as endogenous proteins^27^,^28^,^29^. As other Diaphanous-related formins, FMNL2 is regulated by autoinhibition^14^,^30^, which in this case can be relieved by interaction with the small Rho-family GTPase Cdc42 (ref. 29). Similar observations were recently described for FMNL3 (ref. 28), suggesting that both formins might contribute to the efficiency of protrusion, in particular downstream of Cdc42 signalling. But what are the mechanistic functions of FMNL formins in protrusion?

It is clear that upon activation FMNL2 and FMNL3 employ their proline-rich FH1- (formin homology 1) domain and actin binding domains such as FH2 (formin homology 2) or FH2 plus WH2 (WASP homology 2), in case of FMNL3, for stimulating actin filament assembly^31^,^32^. Moreover, FMNL2 can enhance processive actin filament elongation in the presence of profilin *in vitro*^29^.

This notion and the observed reduction of protrusion upon RNAi-mediated FMNL2 knockdown led to a model in which FMNL formins—possibly in analogy to Ena/VASP family actin polymerases^33^,^34^—would promote elongation of lamellipodial actin filaments nucleated by Arp2/3 complex^3^,^29^. So following this model, lamellipodial actin assembly would result from the coordinated branching and FMNL formin-dependent elongation of actin filaments, with slow but persistent protrusion if Arp2/3-mediated branching dominated, and rapid, unstable protrusion if formin-mediated elongation dominated^3^. Here, we explored this hypothesis by combining comparative analyses of the biochemical activities of FMNL2 and FMNL3 with examination of the consequences of FMNL2/3 loss of function in migrating melanoma and fibroblast cells.

We show that FMNL2 and FMNL3 display moderately divergent activities in actin nucleation and elongation *in vitro* and partially overlapping functions in lamellipodia protrusion. However, simultaneous functional interference with FMNL2 and -3 reduces the width of lamellipodia as well as the actin filament density and frequency of microspike bundle formation within them, with Arp2/3-dependent filament branching staying unchanged. Nevertheless, this treatment strongly compromises the pushing forces exerted by these structures in B16 cells. Consequently, FMNL2/3 removal coincides with compromised migration of both B16 cells and fibroblasts, and reduced capability of the latter to move in high viscosity. We conclude that FMNL formin-generated filaments in lamellipodia operate in addition to Arp2/3 complex-dependent branching to strengthen these structures for promoting effective protrusion and migration.

## Results

### FMNL2 and FMNL3 display common and divergent activities

**Figure 1:**
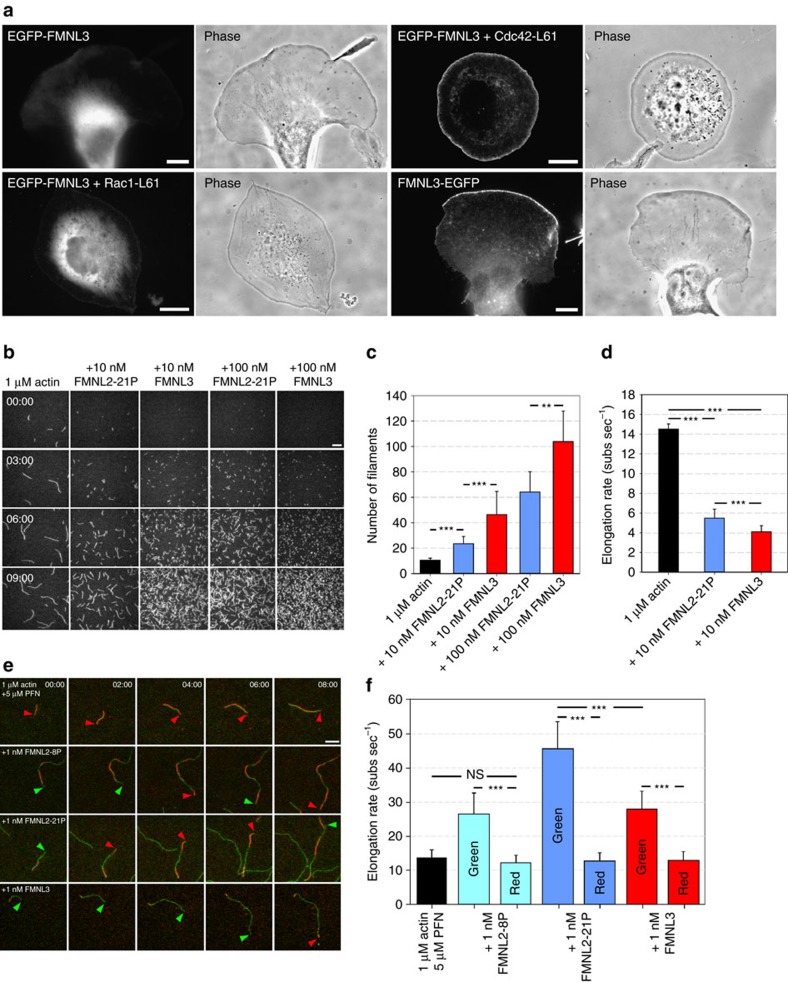
FMNL3 is regulated like FMNL2, but displays diverging actin assembly properties. (**a**) Fluorescence and phase-contrast images of B16 cells expressing EGFP-FMNL3, FMNL3-EGFP or EGFP-FMNL3 co-transfected with constitutively active Cdc42 (Cdc42-L61) or Rac1 (Rac1-L61), as indicated. As shown previously with FMNL2 (ref. 29), EGFP-FMNL3, which lacks N-terminal myristoylation, is activated to target to lamellipodia by active Cdc42 but not Rac1, whereas C-terminally tagged FMNL3 is fully regulated in the absence of additional signals. Scale bars, 10 μm. (**b**) Time-lapse micrographs of TIRF microscopy (TIRF-M) assays for determination of nucleation activities. Polymerization of 1 μM G-actin (10% labelled with ATTO488, 5% labelled with ATTO565) in the absence and presence of FMNL2-21P or FMNL3 at concentrations as indicated. Only red channels are displayed in greyscale, time is in minutes; bar, 10 μm. (**c**) Nucleation activities of FMNL2-21P and FMNL3 in the absence of profilin. Filament numbers from TIRF-M images shown in **b** were counted 3 min after addition of monomeric actin (G-actin). Error bars represent s.d. (**d**) Average elongation rates of actin filaments in the presence of FMNL2-21P or FMNL3 at 10 nM compared to control filaments. Both formins significantly suppressed filament growth in the absence of profilin. Error bars represent s.d. (**e**) Time-lapse micrographs of TIRF-M assays, used for analysis of both elongation and processivity (Supplementary Fig. 4d) for each formin fragment. Polymerization of 1 μM G-actin (10% labelled with ATTO488, 5% labelled with ATTO565) with 5 μM profilin in absence and presence of 1 nM FMNL2-8P, FMNL2-21P or FMNL3. Green arrowheads mark formin-elongated and red arrowheads freely growing filaments. Time, min; bar, 10 μm. (**f**) Quantification of actin filament elongation rates at low formin concentrations (1 nM), allowing distinction between fast, formin-mediated growth and slow, formin-independent growth. Note that FMNL2-21P elongated filaments significantly faster than FMNL3. FMNL2-8P-mediated elongation was slower than that driven by FMNL2-21P, as expected^29^, but comparable to FMNL3. **p<0.01; ***p<0.001 by Mann–Whitney rank sum test.

We previously established a physiologically relevant, direct interaction of the N-terminal regulatory half of FMNL2 with Cdc42 (refs 29, 35). To test whether Cdc42 can relieve the autoinhibition of FMNL3 in migrating B16-F1 cells, as previously found for FMNL2, we expressed EGFP-FMNL3 either alone or in combination with constitutively active Cdc42 or Rac1 (Fig. 1a). In analogy to FMNL2 (ref. 29), EGFP-FMNL3 is cytosolic, partly because EGFP-tagging at the N-terminus blocks its N-terminal myristoylation and because endogenous Cdc42 is insufficient to fully activate the formin. Co-overexpression of Cdc42 activated EGFP-FMNL3, allowing targeting to lamellipodia tips, which was not observed for Rac1 that stimulated lamellipodia without EGFP-FMNL3 accumulation. C-terminal tagging of FMNL3, enabling myristoylation, confirmed the lamellipodium in B16 cells as physiologically relevant site of FMNL3 accumulation (Fig. 1a, Supplementary Movie 1).

We next explored the biochemical activities of FMNL formins and their combined functions *in vivo*, as their distinguished accumulation at the lamellipodium tip where actin assembly takes place^25^ suggests a key role in the formation of these structures. As FMNL1 was completely absent in B16 cells (Supplementary Fig. 1), as expected^22^, we focused on FMNL2 and FMNL3.

The C-terminal halves of both formins constituting the business ends concerning actin filament assembly were compared. As opposed to FMNL3, the C-terminus of FMNL2 could only be expressed as synthetic versions lacking variable numbers of repetitive proline residues within the FH1 domain, termed FMNL2-8P and FMNL2-21P for short and long versions, respectively. Consistent with previous observations^29^ and with other formins^36^, the number of proline residues positively correlated with activity (see below).

In pyrene assays and in the absence of profilin, FMNL3 accelerated actin assembly in a concentration-dependent manner, as reported previously^32^, but this was much less pronounced in case of FMNL2-21P (Supplementary Fig. 2a,b). A similar pattern was observed in the presence of profilin (Supplementary Fig. 3a), although the normalized polymerization slopes peaked at concentrations of about 100 nM in each case (Supplementary Fig. 3b). However, due to the reduced binding of profilin to pyrene-actin^37^, hindering precise quantification of formin-dependent actin assembly in these bulk assays, potential differences in nucleation and/or elongation activities between these formin variants were additionally explored at the single filament level by TIRF microscopy.

Both formins stimulated filament formation in a concentration-dependent manner, but FMNL3 was significantly more potent in the absence of profilin than FMNL2-21P (Fig. 1b, c, Supplementary Movie 2). Increased filament numbers coincided with decreased filament length (Fig. 1b), consistent with these formins slowing down barbed end elongation in the absence of profilin (Fig. 1d). Although profilin addition caused reduced average nucleation of actin filaments by both formins (Supplementary Fig. 4a,b), FMNL3 performed better again in this assay, albeit statistically insignificant. Together, we conclude that FMNL3 nucleates actin filaments more potently than FMNL2. This is also consistent with enhanced induction of filopodia (Supplementary Fig. 5a) upon over-expression of active mutants of FMNL3 (FMNL3-A275E) as compared to active FMNL2 (FMNL2-A272E), both of which were generated in analogy to previously characterized active variants of mDia1 (A256D) (ref. 38) or FHOD (V228E) (ref. 39).

These results also prompted us to test whether FMNL3 might drive nucleation of lamellipodial filaments in the absence of the Rac/WAVE/Arp2/3 complex pathway, for example, in Rac-deficient cells completely lacking lamellipodia^40^. However, neither active FMNL3 (ΔDAD) nor activation of full length FMNL3 by constitutively active Cdc42 restored lamellipodia formation in Rac-null cells, in spite of prominent targeting of these FMNL3 variants to filopodia tips or concave cell edges formed in between them (Supplementary Fig. 5b).

Profilin-dependent filament elongation by both formins was compared at two concentrations. At 10 nM, FMNL2-21P almost tripled average elongation rates in the presence of profilin whereas FMNL3 was less effective in this assay (Supplementary Fig. 4c). We also tested formin variants at 1 nM concentration and with differentially tagged actin variants (see Methods), which allowed directly distinguishing spontaneous from formin-mediated actin filament elongation based on fluorescence colour of incorporated actin (Fig. 1e,f, Supplementary Movie 3). Here, measured elongation rates observed with FMNL2-21P rose up to ∼45 subunits per second (at ∼1 μM profilactin), whereas rates observed with FMNL3 or the truncated FMNL2-8P at identical conditions did not exceed 30 subunits per second (Fig. 1f). Little difference was observed between FMNL2-21P and FMNL3 concerning processive elongation, but FMNL2-8P was clearly less active in this assay (Supplementary Fig. 4d), indicating that the number of proline stretches within FH1 can contribute to the persistence of formin binding at the barbed end.

Together, FMNL2 is more effective in elongating actin filaments than FMNL3, opposite to what was observed for nucleation. These data suggest that FMNL2 and FMNL3 functions *in vivo* are not fully overlapping, as exemplified for instance by pronounced induction of filopodia by FMNL3 as compared to FMNL2 (Supplementary Fig. 5a).

### FMNL2 and -3 variably contribute to lamellipodium protrusion

**Figure 2:**
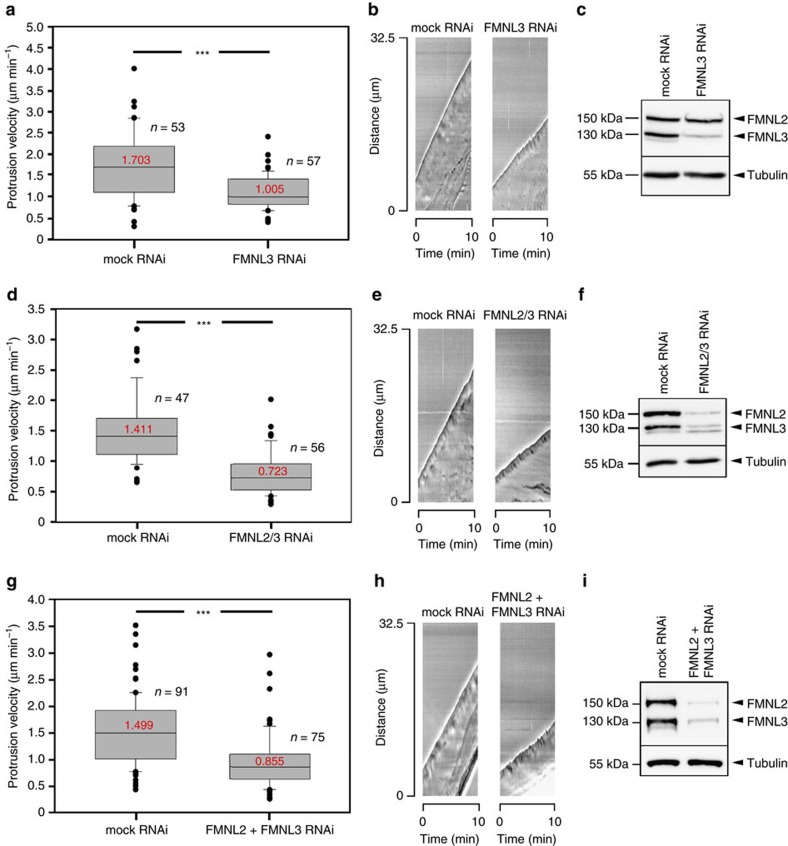
Depletion of FMNL2 and -3 modifies efficiency of lamellipodium protrusion. Average protrusion velocities of B16-F1 cells upon knockdown of FMNL3 alone (**a**–**c**) or two alternative approaches of simultaneous knockdown of FMNL2 and FMNL3, either using one plasmid capable of knocking down expression of both proteins (FMNL2/3 RNAi; **d**–**f**) or two plasmids targeting both messages specifically (FMNL2+FMNL3 RNAi; **g**–**i**). (**a**,**d**,**g**) Box and whiskers plots with median values given in red, boxes including 50% (25–75%) and whiskers 80% (10–90%) of all measurements; outliers are shown as dots. *n*, number of cells analysed. Measurements were aided by assembling kymographs (**b**,**e**,**h**) of phase contrast movies. Knockdown efficacies were documented for each individual experiment, with representative western blots shown in **c**,**f**,**i**. Tubulin was used as loading control. ***p<0.001 by Mann–Whitney rank sum test.

FMNL2 knockdown reduces lamellipodium protrusion speed in migrating B16-F1 melanoma cells by 24% (ref. 29). To test for the relative relevance of FMNL2 versus FMNL3 in lamellipodia formation, in particular in the context of their divergent biochemical activities described above, we transiently silenced expression of FMNL3 alone (Fig. 2a–c) or FMNL2 and FMNL3 by RNA interference (Fig. 2d–i). Double knockdown was achieved by expression of one shRNA mediating knockdown of both proteins (Fig. 2d–f) or of combined shRNAs mediating their knockdown individually (Fig. 2g–i, Supplementary Movie 4). Both approaches caused protrusion rates to be reduced to roughly half of the rates observed in mock RNAi-treated cells, but knockdown of FMNL3 had effects much stronger than previously seen for FMNL2 (reduced by ∼41%; Fig. 2a–c). This was counterintuitive, at least at first glance, as promotion of protrusion was previously proposed to be mediated by actively elongating actin filaments^3^,^29^, an activity assigned to FMNL2 rather than FMNL3. Notwithstanding this, our analyses established both FMNL2 and FMNL3 to contribute to protrusion, albeit in a non- or at best partially redundant fashion, consistent with their distinct biochemical activities observed *in vitro*. Importantly, reduction of lamellipodium protrusion in these cells also affected migration efficiencies, again with FMNL3 contributing more substantially to the process (Supplementary Fig. 6a,b) than FMNL2 (ref. 29). In contrast, the persistence of lamellipodium protrusion, assessed as average time until B16 lamellipodia underwent collapse or retraction, as well as the directionality of migration were unchanged upon FMNL formin knockdown (Supplementary Fig. 7a,b), indicating that the observed reduction of migration was directly coupled to limited protrusion.

### FMNL2/3 knockdown affects various lamellipodial parameters

It is commonly assumed that protrusion rates of lamellipodia are regulated by the polymerization of filaments within the network, but also by their stiffness or anchorage of the network to further proximal regions or the underlying substratum^3^,^41^,^42^. FMNL2 was previously proposed to contribute to protrusion by accelerating the elongation of actin filaments branched by Arp2/3 complex^3^,^29^. To explore this possibility experimentally, also in light of enhanced nucleation observed for FMNL3, we first determined polymerization rates of lamellipodial actin networks relative to protruding front and underlying substratum^25^. The speed of rearward translocation of lamellipodial actin networks and hence their rate of polymerization has previously been visualized and quantified essentially by two experimental approaches: (i) bleaching or photoactivation of networks co-incorporating tagged actin variants^25^,^43^ or (ii) tracking of actin speckles or of fluorescent inhomogeneities of factors bound to these actin networks^44^,^45^,^46^. Here, we have employed both approaches. Unexpectedly, however, rates of lamellipodial actin network translocation were much less prominently reduced in FMNL2/3 double-knockdown cells as compared to controls than the average protrusion rates of the same cells, irrespective of whether network translocation rates were determined by fluorescence recovery of photobleaching (FRAP) of EGFP-β-actin (Supplementary Fig. 8a,b, Supplementary Movie 5) or, alternatively, by following translocation of EGFP-lifeact speckles (Supplementary Fig. 8c,d). The lifeact approach was also chosen as control, as EGFP-β-actin overexpression modestly increased protrusion rates irrespective of RNAi treatment (Supplementary Fig. 8b, left panel), for unknown reasons but consistent with previous observations^47^. Nevertheless, actin polymerization rates of lamellipodia networks were identical in cells expressing EGFP-actin versus EGFP-lifeact, irrespective of formin knockdown, reassuring the conclusion of unchanged actin network assembly rates in these experimental conditions (Supplementary Fig. 8b, d, right panels). These data revealed that reduction of protrusion upon FMNL2/3 knockdown cannot be explained by reduced polymerization rates of underlying actin networks, as one would expect from current models of lamellipodium protrusion^3^,^42^. More specifically, these observations are inconsistent with the currently prevalent view of actin assembly factors such as FMNL formins to simply accelerate the growth of lamellipodial actin networks by stochastically capturing and elongating F-actin barbed ends generated by Arp2/3 complex-mediated branching^3^,^29^.

**Figure 3:**
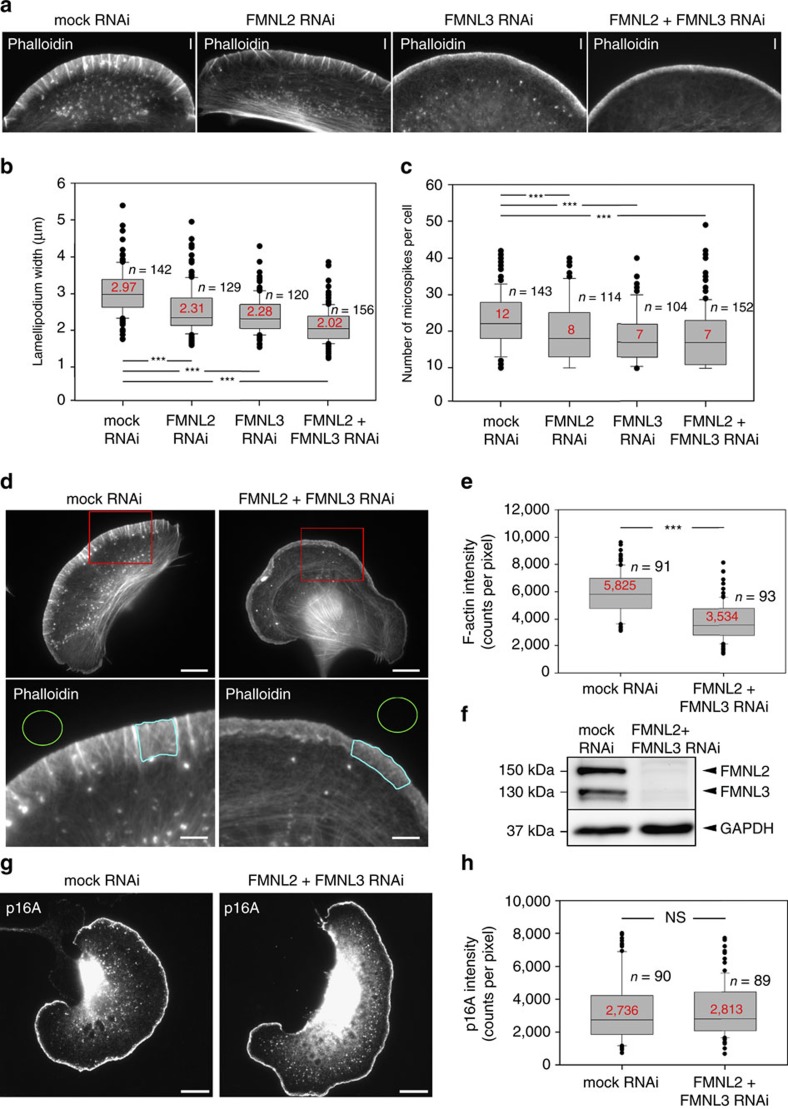
RNAi-mediated knockdown of FMNL2 and -3 reduces lamellipodium width, microspike number and F-actin intensity. (**a**) Representative examples of lamellipodia from mock, FMNL2, FMNL3 or FMNL2+FMNL3 RNAi-treated B16 cells fixed and phalloidin-stained during migration on laminin. Bars, 3 μm. (**b**,**c**,**e**) Quantification of lamellipodium width (μm), microspike number and F-actin intensity in lamellipodia (photon counts pixel^−1^ after background subtraction) in cells treated as in **a**. Results are displayed as box and whiskers plots with median values given in red, boxes including 50% (25–75%) and whiskers 80% (10–90%) of all measurements; outliers are shown as dots. *n*, number of cells analysed. (**d**) Examples of phalloidin-stained B16 cells RNAi-treated as indicated, and employed for quantification of F-actin intensity. Red rectangles in top panels indicate magnified insets shown at the bottom. Areas framed in turquoise depict representative regions within lamellipodia used to measure F-actin intensities, and green circles extracellular regions defined as background. Scale bars in upper and lower panels equal 10 and 3 μm, respectively. (**f**) Representative western blot illustrating RNAi-mediated knockdown efficiency from samples collected in parallel to cells used in **b**,**c**,**e**. (**g**) Immunolabelling of the Arp2/3 complex subunit p16A (ARPC5A) in RNAi-treated cells as indicated. Bars, 5 μm. (**h**) Quantification of Arp2/3 complex intensities in lamellipodia of mock- and FMNL knockdown cells, displayed as described in **b**,**c**,**e**. ***p<0.001 by Mann–Whitney rank sum test.

However, when exploring additional parameters of lamellipodial protrusion and structure, we found that various features of lamellipodia were markedly changed upon FMNL2/3 knockdown. First, lamellipodia in knockdown cells were narrower than in control cells, apparent already in EGFP-actin and lifeact movies (Supplementary Fig. 8, Supplementary Movie 5). This was substantiated by quantification of lamellipodial width upon knockdown of FMNL formins in various combinations (Fig. 3a,b). As lamellipodium width was previously observed to be directly controlled by members of the ADF/cofilin family^48^, thought to sever and thus reduce filament lengths and lamellipodium width^49^, we sought to exclude the observed differences to be explained by changes in ADF/cofilin expression or activity. ADF/cofilin proteins are invariably inactivated by phosphorylation on serine 3, so we quantified levels of phosphorylated, inactive cofilin 1, the largely ubiquitous family member, as well as of total cofilin 1 by western blotting (Supplementary Fig. 9). However, cofilin 1 expression was unchanged upon transient FMNL2/3 knockdown as compared to controls, whereas levels of phosphorylated, inactive cofilin were even increased. These data suggest that formin knockdown-induced lamellipodial narrowing cannot be caused by an increase of ADF/cofilin activity. Interestingly, the apparent decrease in cofilin 1 activity might be caused by compensatory mechanisms, as wild-type levels of cofilin activity should cause even more pronounced lamellipodial narrowing.

We next examined the overall structure of the lamellipodium in mock-treated versus FMNL2/3 knockdown cells, the latter of which appeared less organized, with lower numbers of embedded microspike bundles (Supplementary Fig. 10). Quantification of microspike bundles using phalloidin (Fig. 3c) or its most prominent constituent fascin^50^ (Supplementary Fig. 11) revealed a reduction upon individual or combined knockdown of both FMNL2 and FMNL3.

The observed effects on bundle frequency within the lamellipodium and established functions of these formins in filament formation *in vitro* prompted us to examine the density of lamellipodial actin networks, irrespective of the already apparent overall reduction of filament mass due to lamellipodial narrowing. Interestingly, phalloidin intensities of double-knockdown cells were further reduced to roughly 60% of the levels observed in control cells (Fig. 3d–f). To explore whether filament loss was specific for the formin pathway or also had an impact on Arp2/3 complex-dependent actin network generation, we also stained for incorporation of endogenous Arp2/3 complex into these lamellipodia, which was not reduced upon formin knockdown (Fig. 3g,h). Collectively, these data suggest that although incapable of generating lamellipodia without the Arp2/3-dependent branching pathway (Supplementary Fig. 5b), FMNL formins contribute significantly to mass and structure of lamellipodial actin networks, with potential impact on their mechanical stability and protrusion effectivity aside from their sole role in regulating protrusion rates (Fig. 2). This view was further supported by the observation that FMNL2/3 double-knockdown cells displayed fluctuations of the lamellipodial tip membrane—indicative of inefficient protrusion—much more frequently than mock cells, which consistently protruded in a smooth, continuous fashion in these migration conditions (Supplementary Fig. 6c,d, Supplementary Movie 6). Thus, we sought to assess potential effects of FMNL loss of function on protrusion stability and force.

### Generation and characterization of FMNL-deficient B16 cells

**Figure 4:**
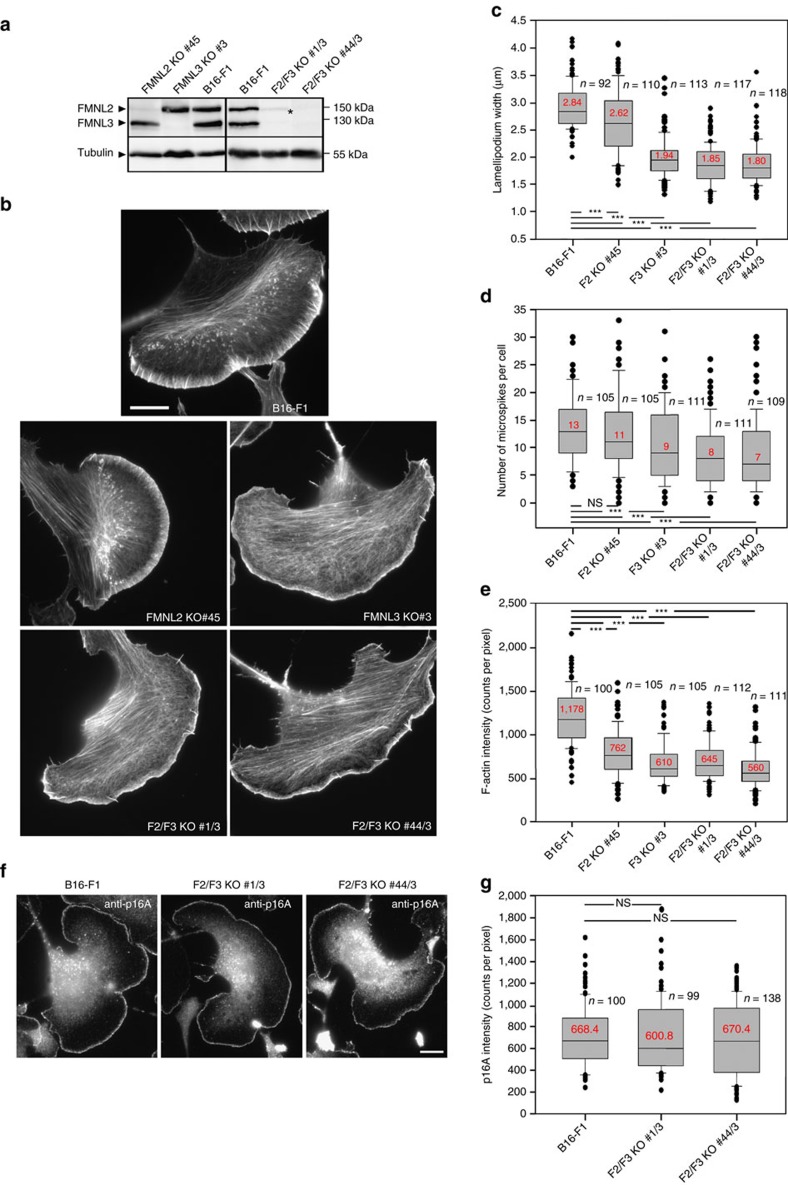
Knockout of *FMNL2/3* recapitulates RNAi phenotypes. (**a**) CRISPR/Cas9-mediated, specific loss of protein expression as documented by western blotting, using FMNL2/3-reactive antibody and tubulin as loading control. Asterisk marks non-specific band. (**b**) Representative cells stained for the actin cytoskeleton with phalloidin harbouring genotypes as indicated. Bar, 10 μm. (**c**–**e**) Box and whiskers plots summarizing the results from respective quantifications of lamellipodium width, microspike number per cell and F-actin intensity done in analogy to what is shown in Fig. 3. (**f**) Immunolabelling of the Arp2/3 complex subunit p16A (ARPC5A) in B16 cells and *FMNL2/3* KO cells as indicated. Scale bar, 10 μm. (**g**) Quantification of Arp2/3 complex intensities in lamellipodia of wild-type and *FMNL2/3 KO* cells. ***p<0.001 by Mann–Whitney rank sum test.

To extend our studies to ultrastructural analyses and force measurements requiring complete absence of FMNL function at the single cell level, which is impossible to achieve per definition by RNA interference, we genetically disrupted *FMNL2* and *FMNL3* genes in B16 cells using CRISPR/Cas9-mediated genome editing. We initially isolated clonal cell populations with disrupted expression of either *FMNL2* or *FMNL3* alone followed by disruption of the respective other gene. In total, one KO for each gene and two independently generated double-KO clones were analysed further (Fig. 4a,b). In general, CRISPR/Cas9-treated cells displayed phenotypes remarkably similar to RNAi-mediated knockdowns, confirming the specificity of either approach. In analogy to RNAi experiments, all formin KO cell populations displayed reduced lamellipodial widths, microspike numbers and lamellipodial F-actin intensities, albeit to variable extents. Loss of *FMNL2* alone generally caused weaker phenotypes than loss of *FMNL3*, and double-KOs on average displayed stronger phenotypes than individual KOs (Fig. 4c–e). Importantly, both *FMNL2/3* double-KO clones also lacked any detectable staining of FMNL protein at the lamellipodium tip (Supplementary Fig. 12), confirming specificity of the antibody employed previously for FMNL2/3 immunolabelling^29^. Reduction of actin filament intensity was particularly evident upon FMNL2/3 loss of function (Fig. 4e), whereas endogenous Arp2/3 complex appeared incorporated at virtually equal levels in the lamellipodia of control versus double-KO cell lines (Fig. 4f,g). Along these lines, a 5-min treatment of B16 control and *FMNL2/3* double-KO cells (F2/F3 KO #44/3) with the Arp2/3 complex inhibitor CK666 (210 μM) reduced lamellipodial width by about one-third for both cell types (Supplementary Fig. 13). These data suggest that sensitivity to interference with Arp2/3 complex function is independent of FMNL formin expression.

The average reduction of lamellipodium width upon loss of FMNL2 and -3 was again not caused by an increase in ADF/cofilin function, as both cofilin 1 levels and activity were not considerably changed in FMNL2/3 double-KO cell lines (Supplementary Fig. 14).

**Figure 5:**
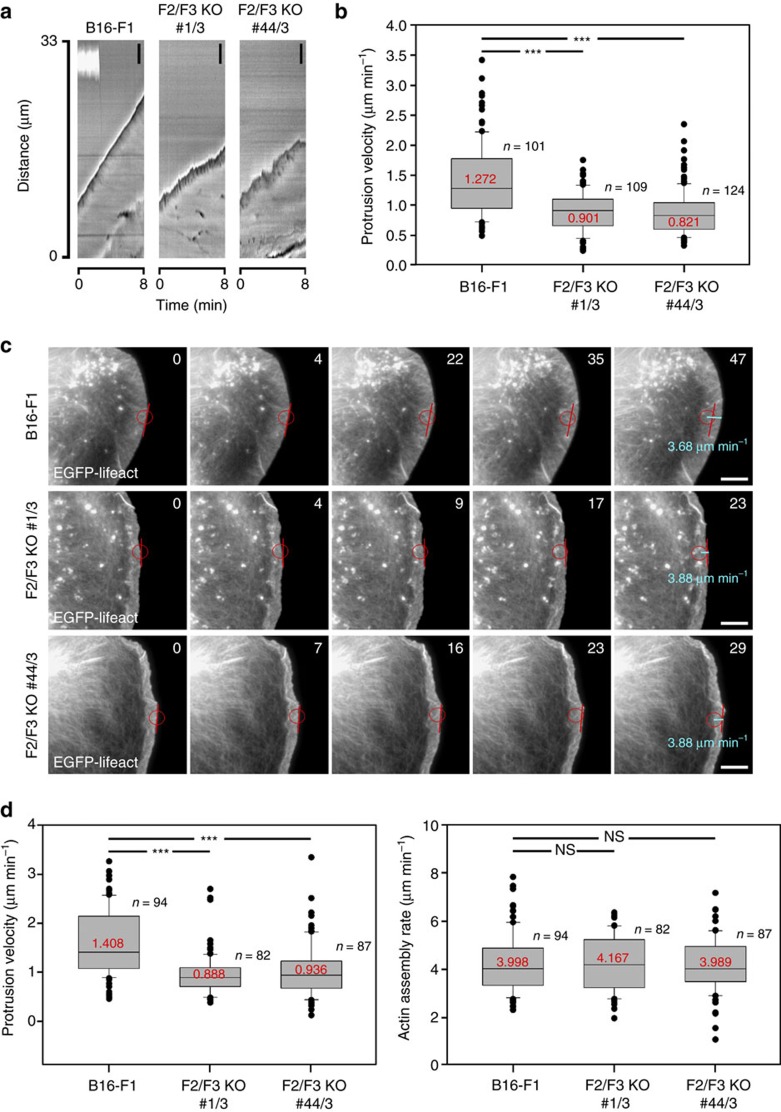
Reduced protrusion in *FMNL2/3* KO is not caused by decreased network polymerization. (**a**) Representative kymographs demonstrating lamellipodial protrusion of *FMNL2/3* knockout cells compared to B16 controls. Note that protrusion in case of F2/F3 KO#1/3 and F2/F3 KO#44/3 is much less smooth as usually seen with control B16 (left panel). Bars equal 3 μm. (**b**) Box and whiskers plots depicting average protrusion velocity (μm min^−1^). Red numbers denote medians of respective data sets; *n* gives the number of cells analysed. (**c**) Time-lapse frames of B16 and respective *FMNL2/3* KO clones transfected with EGFP-lifeact. Images show sections of respective lamellipodia before and during tracking of fluorescence inhomogeneities performed to determine lamellipodial actin assembly rates; for experimental detail, also see legend to Supplementary Fig. 8c. Time is in seconds, bars equal 5 μm. (**d**) Average protrusion velocities (left panel) or actin assembly rates (right panel) of respective cell types expressing EGFP-lifeact. Box and whiskers plots are as shown in **b**. In spite of significant reduction of average protrusion velocities in *FMNL2/3* KO lines (left panel), the rate of lamellipodial actin assembly is almost identical (right panel). ***p<0.001 by Mann–Whitney rank sum test.

Importantly, both double-KO cell lines also displayed significantly reduced protrusion rates (Fig. 5a,b) and migration efficiencies (Supplementary Fig. 15a,b), and were phenocopies of FMNL2/3 double-RNAi cells concerning increased frequencies of cells displaying fluctuating protrusions (Supplementary Fig. 15c,d). Correlation analysis of protrusion features of pooled double-KO and wild-type B16 lamellipodia revealed a clear correlation between protrusion velocity and lamellipodial width in these experimental conditions, and showed that the observed fluctuation behaviour preferentially occurred below specific threshold levels for both parameters (see colour code in Supplementary Fig. 15e). None of these lamellipodial phenotypes were caused by evident changes in adhesion patterns, as staining the focal adhesion component vinculin followed by machine-aided quantification revealed no changes in the different cell lines, neither in average size nor number of adhesions (Supplementary Fig. 16a–c). The relative percentage of focal versus nascent adhesions, with the latter being defined as <0.2 μm^2^ in size, was also unchanged upon FMNL2/3 removal (Supplementary Fig. 16d). We thus conclude that lamellipodial phenotypes are not caused by changes in adhesion or traction forces, but instead by more direct means.

However, combined genetic disruption of FMNL2 and -3 in this cell type did not reduce rates of lamellipodial actin network assembly (Fig. 5c,d), in spite of significant reduction of protrusion analysed in parallel, again consistent with RNAi data (see above and Supplementary Fig. 8).

Altogether, these results set the stage for examining the mechanistic reasons for the reduced protrusion phenotype observed, including analysis of force development of respective lamellipodia upon FMNL formin loss of function, as well as ultrastructural organization of underlying actin networks.

### FMNL removal abrogates force generation by B16 lamellipodia

**Figure 6:**
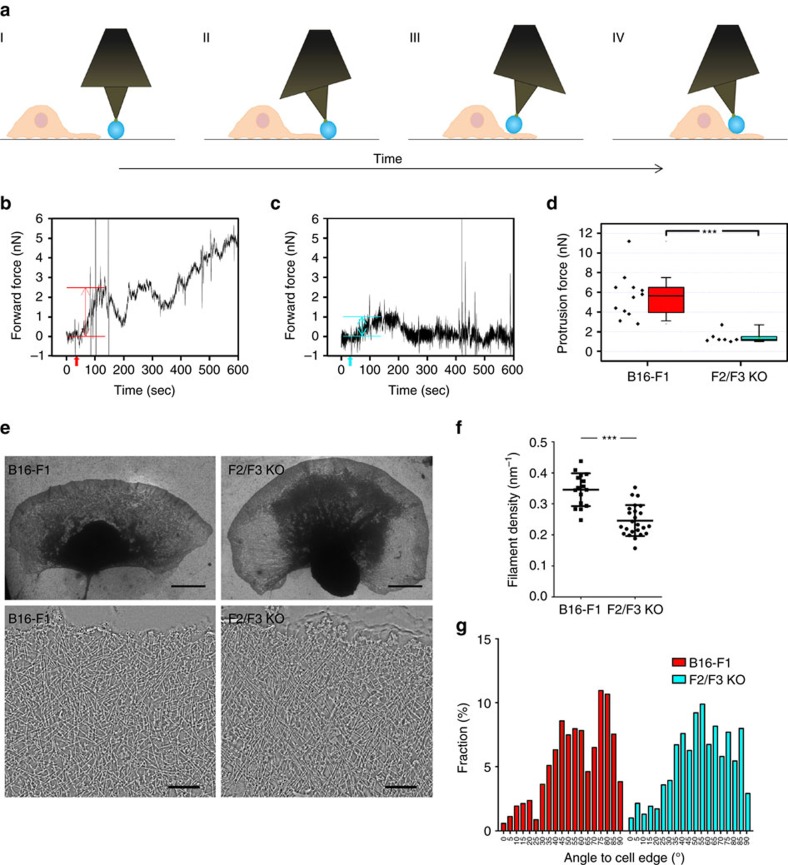
Loss of FMNL2/3 affects ultrastructure and force generation of lamellipodia. (**a**) Schematic illustration of experimental stages. A modified cantilever is placed at a preset force of 1 nN in front of and perpendicular to a migrating cell (I). The cell pushing against the bead causes a lateral deformation of the cantilever (II). If the cell is strong enough to migrate further, the cantilever is lifted and laterally deflected into the opposite direction due to retrograde flow of lamellipodium and lamella (III). Finally, the cell body causes a much higher deflection of the cantilever (IV). (**b**) Measurement of a B16 wild-type cell representative for the force deflection curve typically derived from the series of events depicted in **a**. The red arrow on the time axis indicates first contact between cell and bead. The red double-arrow shows the amount of protrusion force exerted by the lamellipodium. The rest of the curve is explained by stages depicted in **a** (experimental stages III and IV). (**c**) Representative measurement of *FMNL2/3* double-knockout cell. Turquoise arrow on time axis and double-arrow indicate first cell/bead contact and protrusion force exerted by the KO-cell, respectively. Commonly, KO cells were not strong enough to lift the bead onto the lamellipodium, but instead frequently changed direction of migration. (**d**) Summary of protrusion force experiments shown as box and whiskers plots. (**e**) Overview images of transmission electron micrographs of representative wild-type (B16-F1) or *FMNL2/3* double-KO cells, as indicated (top) and 5.5 μm electron tomography slides showing respective actin filament network at the leading edge (bottom). Scale bars, 10 μm (top panels), 100 nm (bottom panels). (**f**) Quantification of projected filament density from four different regions per analysed cell. Data are displayed as arithmetic means±s.d. (**g**) Histogram of angles subtended by filaments to the leading edge (90° corresponding to filaments perpendicular to the leading edge). ***p<0.001 by Mann–Whitney rank sum test.

To determine lamellipodial forces that can be exerted against obstacles, beads mounted on atomic force microscopy (AFM) cantilevers were positioned right in front of protruding double-KO (F2/F3 KO #44/3) or B16 control lamellipodia. Protrusion was observed by phase-contrast microscopy, and vertical and lateral cantilever deflections were recorded over time (Fig. 6a and legend). Deflections were computed into forward forces, plotted over time (Fig. 6b,c) and used for determination of protrusion forces (Fig. 6d). Twelve wild-type B16 lamellipodia exerted an average protrusion force of 5.6±2.3 nN, which was reduced by 75% to 1.4±0.6 nN in *FMNL2/3* null cells (*n*=7). This average reduction is even underestimated, since four cells clearly touched the beads, but the forces exerted by them were below the resolution of the cantilevers employed, so we excluded them from further analysis. Furthermore, upon extended lamellipodial-bead contact, KO cells frequently failed to move their cell bodies towards the bead, as opposed to control cells (Fig. 6c), and turned away in another direction (compare force deflection curves in Fig. 6b,c). Together, these data clearly reveal a remarkable loss of ability to exert lamellipodial forces upon FMNL loss of function, although effects on actin networks—at least at the light microscopy level—appeared comparably modest. This phenotype and the lack of changes in traction forces (see above) prompted us to explore the ultrastructural organization of FMNL-deficient lamellipodia by electron tomography of negatively stained samples.

### FMNL2/3 KO causes ultrastructural changes in lamellipodia

Reduction in force development of lamellipodial networks could be theoretically caused by multiple parameters, including numbers and angles of filaments abutting the protruding membrane. As opposed to Arp2/3 complex inhibition, which eliminated actin networks reminiscent of lamellipodia and actin network treadmilling^51^, FMNL formin-deficient lamellipodial networks polymerized actin in a treadmilling fashion (Fig. 5c,d) and looked similar to controls at first glance (Fig. 6e). However, computer-assisted assessment of filament numbers at the lamellipodium tip (within a region of approximately 1 μm from the edge) confirmed a clear and statistically significant reduction in filament mass (Fig. 6f), consistent with our observations using fluorescence microscopy (Fig. 4e).

In addition, both control and knockout lamellipodia displayed a wide range of filament angles abutting the protruding front, with two clear peaks at around 45° and 80° relative to the edge in controls (Fig. 6g). In contrast, the distribution of filament angles in FMNL2/3-deficient B16 lamellipodia was slightly different, as the two peaks were less clearly separated and fractions of filaments with close to orthogonal angles underrepresented as compared to controls (Fig. 6g). Together, in spite of comparably subtle changes in ultrastructural organization of freely protruding filament networks, FMNL formin removal causes clear changes in filament mass and orientation in these structures, possibly rendering them less prone to exert required pushing forces when confronted with obstacles.

### *FMNL2/3* KO in fibroblasts impairs lamellipodia and migration

**Figure 7:**
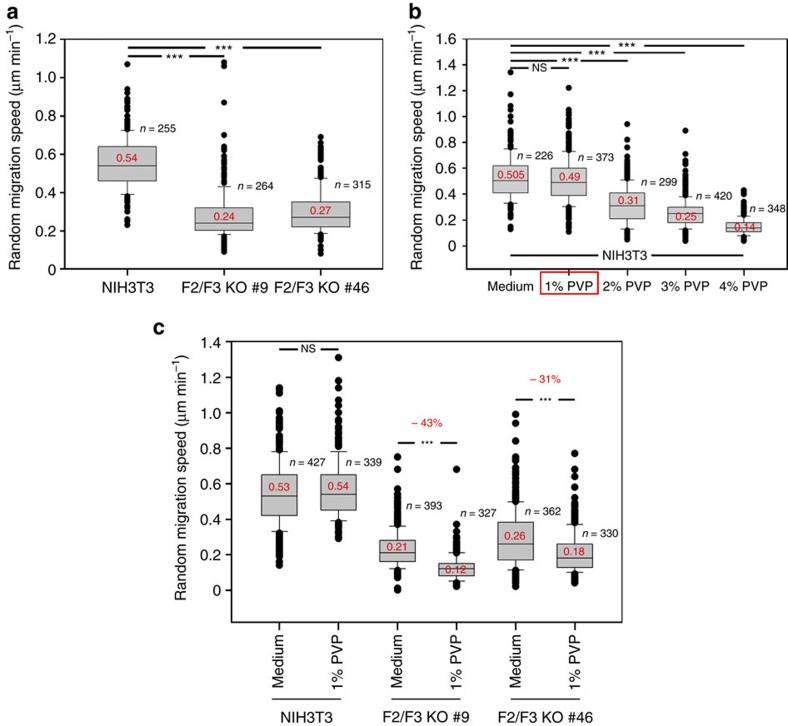
Depletion of FMNL2/3 in fibroblasts recapitulates migration defects. (**a**) Box and whiskers plots summarizing quantification of random migration rates (μm min^−1^) of double-knockout clones and wild-type NIH 3T3 cells as indicated. (**b**) Quantification of random migration speed (μm min^−1^) of NIH 3T3 cells that were either migrating in regular medium or medium supplemented with different concentrations of polyvinylpyrrolidone (PVP) as indicated in the figure. Increase of PVP concentrations linearly correlates with decreased rates of random migration. As 1% PVP did not significantly affect migration of wild-type NIH 3T3 fibroblasts, this concentration was chosen for comparison with *FMNL2/3* KO clones (red rectangle). (**c**) Box and whiskers plots summarizing quantification of random migration rates (μm min^−1^) of double-knockout cell clones #9 and #46 as well as wild-type NIH 3T3 determined in regular medium versus medium of slightly increased viscosity (1% PVP), as indicated. Note the differential effect observed in medium of slightly increased viscosity (1% PVP) in KO clones versus wild-type cells (reduction of migration by 43% caused by 1% PVP in case of F2/F3 KO #9 and by 31% in case of F2/F3 KO #46). ***p<0.001 by Mann–Whitney rank sum test.

B16-F1 melanoma cells are a widely used model system of migration and lamellipodium protrusion^19^,^42^, but to confirm the general relevance of our observations, we extended our studies to a commonly used fibroblast cell line. CRISPR/Cas9-treatment of NIH 3T3 cells allowed us to generate independent lines null for FMNL3 and displaying very low gene dose of FMNL2, the latter being present with multiple distinct alleles (see Methods). Remarkably, FMNL2/3-depleted NIH 3T3 cell clones (Supplementary Fig. 17a,b) formed lamellipodia with moderately reduced widths (Supplementary Fig. 17c), harbouring significantly diminished actin filament intensities (Supplementary Fig. 17d), again with unchanged Arp2/3 complex incorporation (Supplementary Fig. 17e,f). These phenotypes were fully compatible with those established for RNAi- or CRISPR/Cas9-treated B16 cells (see above). Although general adhesion patterns were again highly similar in control versus FMNL2/3-depleted NIH 3T3 cells (Supplementary Fig. 18), migration rates of these fibroblasts were reduced to approximately half of controls (Fig. 7a). As membrane ruffling activity common to migrating fibroblasts hampered AFM measurements, we explored migration of these cells through media with variable viscosity (Fig. 7b,c), which linearly oppose cell motion by Stokes friction. Consistently, increasing concentrations (up to 4%) of the inert, water-soluble polymer polyvinylpyrrolidone (PVP), a thickening agent used previously for increasing the viscosity of tissue culture media^52^, linearly decreased the migration of control NIH 3T3 fibroblasts (Fig. 7b). Moreover, although control fibroblasts displayed no detectable defect in migrating through medium containing 1% PVP, this concentration was sufficient to significantly impair the migration of FMNL2/3-depleted fibroblasts (Fig. 7c). We thus conclude FMNL2/3 loss of function to significantly lower the ability of cells to cope with counteracting forces during migration, as exemplified by employing medium of increased viscosity.

## Discussion

It is well established that the Rac/WAVE/Arp2/3 complex pathway is essential for generation and maintenance of lamellipodia^6^,^8^,^10^,^11^,^40^,^51^,^53^,^54^,^55^,^56^. Much less is known about how actin filament elongators such as formins and Ena/VASP family proteins contribute to protrusion and thus lamellipodia-dependent migration^12^,^14^,^57^. In the current view, Arp2/3 complex-mediated branching of the network provides stability and persistence of protrusion^3^,^41^,^42^, whereas protrusion rate is thought to derive from actin filament elongation by polymerases located at the tip membrane, such as FMNL formins^3^,^12^. According to this model, FMNL-deficient lamellipodia would protrude slowly but persistently, and constitute Arp2/3-dependent networks capable of resisting counteracting force. In contrast, high formin activity would stimulate rapidly growing lamellipodia, with increased elongation and decreased branching activity as well as lamellipodial stability. Hence, increased formin activity and thus lamellipodial actin filament elongation should also coincide with decreased, average Arp2/3 complex incorporation. Clearly, our data call for revision of this view.

**Figure 8:**
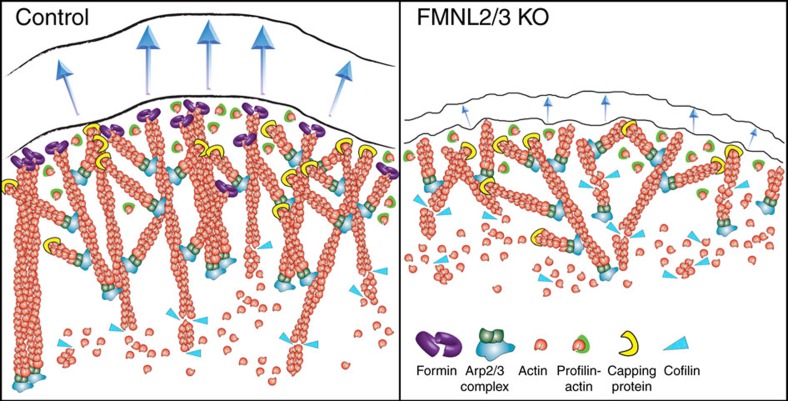
Model summarizing FMNL2/3 loss of function phenotype. Lamellipodial actin filament networks are generated and turned over by combined activities of filament assembly factors (Arp2/3 complex and formins), disassembly factors such as cofilin and by capping protein. As opposed to the branching activity of Arp2/3 complex, lamellipodial formins FMNL2 and FMNL3 are not essential for the generation of lamellipodial actin networks, but proposed to generate specific subsets of filaments contributing to the density and mechanical stability of lamellipodia, essential for effective protrusion and lamellipodial force generation (*FMNL2/3* KO) during migration.

Consistent with previously published data on FMNL2 knockdown^29^, functional interference with FMNL3 or both FMNL2 and -3 reduced protrusion, but not due to decreased rates of actin network polymerization, as previously assumed^3^,^29^. Instead, we conclude that reduction of protrusion was accompanied and caused by loss of specific, formin-dependent filaments, contributing to sufficient lamellipodial filament mass and bundling activity, and to the filament population pushing the tip membrane at close-to-orthogonal angles relative to the front (see Fig. 8). Remarkably, FMNL formin-deficient lamellipodia were weak and unable to exert pushing forces comparable to formin-filament containing controls, although Arp2/3 complex incorporation was quantitatively unchanged as compared to control cell lamellipodia. We note that it is impossible at present to formally exclude that observed phenotypes are not or not entirely connected; hence, more indirect effects potentially caused by FMNL formin removal such as the strength of the lamellipodium/lamella connection and/or force transmission from the lamellipodium to the lamella behind could also contribute to generating fluctuating protrusions and loss of force development. However, we consider the ultrastructural changes within lamellipodia themselves as more likely to impact the forces exerted by these structures, but to confirm this directly, future experimental efforts, including exciting recent developments such as high throughput cryo-tomography of both thin lamellipodia and thicker cellular structures such as the lamella might be required^58^. In any case, our data also suggest that branched dendritic networks alone without the action of formins or additional polymerases will not be efficient in counteracting the forces needed for effective protrusion, as for instance squeezing of lamellipodia-like structures through narrow pores or in between cells in 3D migration *in vivo*. Consistent with this view, overexpression of both FMNL2 and FMNL3 formins was previously found to correlate with invasion and metastasis of colorectal carcinoma^59^,^60^.

Instead of promoting the collective elongation of all filaments within the network, we conclude FMNL2/3 to nucleate and elongate individual filaments, the presence of which is essential for rapid and smooth protrusion as well as efficient cell migration. Thus, our data also imply that in spite of homogenized rates of polymerization at the membrane, lamellipodial networks cannot be simply viewed as sole mass of homogenous filaments, but instead as collection of branched and unbranched filament populations of various kinds all harbouring specific features. More specifically, although filament loss and thus structural considerations alone could potentially explain observed phenotypes, accompanied loss of filament population-specific or network geometry-sensitive actin-binding proteins such as crosslinkers could also account, at least in part, for observed effects. Notwithstanding this, the FMNL-generated, lamellipodial filaments studied here apparently not only contribute to average network mass, but also to filament bundling, and hence stabilization and force development of these actin networks built downstream of Rac, but co-regulated by Cdc42.

## Methods

### Cell culture and transfections

B16-F1 mouse melanoma cells (ATCC CRL-6323) were grown in DMEM (4.5 g l^−1^ glucose; Invitrogen, Germany) with 10% FCS (PAA Laboratories, Austria) and 2 mM glutamine (Thermo Fisher Scientific), and transfected using peqFECT transfection reagent (PeqLab). Three micrograms DNA in total and 6 μl peqFECT reagent were used to transfect cells in 10 cm dishes overnight. NIH 3T3 fibroblasts (ATCC CRL-1658), Raw macrophages (ATCC TIB-71), J774 macrophages (ATCC TIB-67) and *Rac1*^*−/−*^ MEFs^40^ were grown in DMEM (4.5 g l^−1^ glucose), 10% FCS (Sigma), 2 mM L-glutamine, 1% non-essential amino acids and 1 mM sodium pyruvate. Vero (ATCC CCL-81) and MDCKI (ATCC CCL-34) epithelial cell lines were maintained in DMEM (4.5 g l^−1^ glucose), 10% FCS (Sigma) and 2 mM L-glutamine. DU-145 (ATCC HTB-81) and LoVo (ATCC CCL-229) were cultivated in RPMI 1640 medium (Gibco), 10% FCS (Sigma), 2 mM L-glutamine, 1% non-essential amino acids and 1 mM sodium pyruvate. All cells were incubated at 37 °C in the presence of 7.5% CO_2_.

### DNA constructs

EGFP-C1, -C2, -C3 and EGFP-N1, -N2, -N3 vectors used for cloning as well as EGFP-tagged human β-actin were purchased from Clontech Inc. (Mountain View, CA, USA). For cloning of FMNL3 cDNA, a FMNL3ΔN construct was generated using IMAGE Clone IRAKp961L0355Q (imaGenes, Germany) encoding residues 63–1028 of the murine FMNL3 sequence. For generation of FMNL3 full length, the N-terminus (aa 1–103) was amplified from mouse B16 cDNA and fused to the FMNL3ΔN sequence by using an internal BspEI restriction enzyme site. For generation of EGFP-tagged FMNL3, the full-length cDNA was fused into EGFP-C3 using XhoI/BamHI restriction enzymes. Subsequently, full-length FMNL3 was amplified without stop codon using respective primers, subcloned into EGFP-N3 and named FMNL3-EGFP. Truncated variants such as EGFP-FMNL3ΔDAD were generated by PCR with respective primers using EGFP-FMNL3 as template. For generation of FMNL2 cDNA and EGFP-FMNL2ΔDAD, see ref. 29. EGFP-FMNL2-A272E and EGFP-FMNL3-A275E were generated by site-directed mutagenesis using Qiagen site-directed mutagenesis kit employing forward primer 5′-GTCTTAGAACTGTTGGCAGAGGTTTGTCTTGTCAGAGGCG-3′ (in case of FMNL2) and 5′-TGGAGCTGCTGGCAGAGGTGTGTTTGGTGCGG-3′ (in case of FMNL3), and the respective complementary sequences as reverse primers. For generation of FMNL1 expression constructs, the cDNA of full-length FMNL1 (isoform 1, residues 1–1100), UniProt accession number O95466, was purchased as synthetic expression construct from GeneArt (Life Technologies). For EGFP-tagging, the full length sequence was amplified using forward primer 5′-GAGGAATTCATGGGCAATGCTGCCGG-3′ and reverse primer 5′-GAGGGATCCCTAGTGGTGGTGATGATGG-3′ harbouring a stop codon, and ligated into EGFP-C2 using EcoRI and BamHI restriction enzymes. All constructs were sequence verified.

PRK5-myc-Cdc42-L61 and pRK5-myc-Rac1-L61 were kind gifts from Laura Machesky (Beatson Institute, Glasgow, UK). EGFP-lifeact was kindly provided by Roland Wedlich-Söldner (University of Münster, Germany).

Constructs for protein expression were prepared as follows: the C-terminal half of human FMNL3 (residues 498–1028; NCBI reference: NP_035841.1) was amplified by PCR from cDNA using primers 5′-CGCGGATCCATCCCACCCTCTGACTTGGACCTG-3′ (forward) and 5′-CGCGTCGACCTAACAGTTTGACTCGTCATGGTG-3′ (reverse), and ligated into pGEX-6P-1-SNAP expression vector^61^ using BamHI and SalI-restriction sites. The C-terminal half of human Drf3 (mDia2; residues 555–1110) was amplified with primers 5′-GCGGAATTCCCAGCTGATTGTAATATTCCTTTG-3′ (forward) and 5′-CGCGTCGACTTATAAATACGGTTTATTACCATGGTT-3′ (reverse), and inserted into EcoRI and SalI-sites of pGEX-6P-1-SNAP. The FMNL2-8P fragment (residues 566–1092, containing eight consecutive proline residues^29^) was subcloned into the SalI- and NotI-sites of pGEX-6P-1-SNAP. The N-terminus of FMNL2-21P was slightly extended in sequence (corresponding to residues 548–1092, containing 21 consecutive proline residues) using primers 5′-GCGGTCGACGTTACCCCGCCGATGCCTCCGCCC-3′ (forward) and 5′-CGCGCGGCCGCTCACATTGTTATTTCGGCACCATTAAC-3′ (reverse), again followed by ligation into SalI- and NotI-sites of pGEX-6P-1-SNAP.

### Protein expression and fluorescence labelling

C-terminal halves of formins were expressed in fusion to both GST and SNAP-tag for purification and potential fluorescent labelling, respectively. GST-tagged SNAP-FMNL2-8P/21P (FMNL2-8P/21P), SNAP-FMNL3 (FMNL3) and SNAP-Drf3 (Drf3) were expressed in *Escherichia coli* strain ArcticExpress after induction with 0.7 mM IPTG at 12 °C and OD 2. After 20 h, the respective GST-tagged proteins were purified by affinity chromatography using glutathione-conjugated agarose (Sigma-Aldrich). As opposed to previous studies using GST-tagged FMNL2 (refs 29, 35), the GST-tag was subsequently cleaved off by incubation with PreScission protease (GE Healthcare) overnight, and uncleaved GST-SNAP-formin fragments and GST were removed by a second affinity chromatography step with glutathione-agarose. Proteins were further purified by size exclusion chromatography on an Äkta Purifier System equipped with a HiLoad 26/60 Superdex S200 column (GE Healthcare). Fractions containing formin fragments were pooled, dialysed against storage buffer (150 mM KCl, 1 mM DTT, 60% glycerol and 20 mM HEPES pH 7.4) and stored at −20 °C. All assays were performed with unlabelled, SNAP-tagged FMNL fragments, since labelling with benzylguanine-coupled fluorescent probes (SNAP Surface 488/549, New England Biolabs) interfered with formin activity. Expression and purification of untagged human profilin I (PFN) was performed by polyproline affinity chromatography. Of note, the inhibition of spontaneous actin assembly in pyrene-assays seen previously with the GST-tagged C-terminus of FMNL2 (ref. 29) was not observed with SNAP-FMNL2, indicating that additional dimerization through GST might influence the capability of formin dimers to shield barbed ends. All additional experiments were thus performed with SNAP-tagged formin variants.

Rabbit skeletal muscle actin was extracted and purified from acetone powder using standard procedures. Fractions of G-actin were labelled with ATTO 565 maleimide (ATTO-TEC) and with N-(1-Pyrene)maleimide (Life Technologies) at Cys374 or with ATTO 488 NHS-ester (ATTO-TEC) at lysine residues following standard procedures.

### Pyrene-actin polymerization assays

Polymerization of 2 μM rabbit skeletal muscle G-actin (5% pyrene-labelled) was initiated with 1 × KMEI-polymerization buffer (50 mM KCl, 1 mM MgCl_2_, 1 mM EGTA and 10 mM imidazole, pH 7.0) and in the presence of various concentrations of FMNL or Drf3 fragments with or without 10 μM PFN, and was monitored in 96-well plates by a Synergy 4 fluorescence microplate reader (Biotek). Maximum slopes of polymerization curves were determined by linear regression and averaged, *n*=4. The slopes were normalized and plotted against the concentration of the respective formin construct.

### TIRF microscopy

For total internal reflection fluorescence microscopy, FMNL fragments (1, 10 or 100 nM final concentration) and PFN (5 μM final concentration) were first pre-diluted in 1 × TIRF buffer (20 mM imidazole pH 7.4, 50 mM KCl, 1 mM MgCl_2_, 1 mM EGTA, 20 mM β-mercaptoethanol, 0.5 mM ATP, 15 mM glucose, 2.5 mg ml^−1^ methylcellulose (4,000 cP), 20 μg ml^−1^ catalase, 100 μg ml^−1^ glucose-oxidase).

The assays were started by adding G-actin (1 μM final concentration, 10% ATTO488-labelled at lysine residues, 5% ATTO565-labelled at Cys374) and flushing the mixtures into mPEG-silane (Laysan Bio)-coated flow chambers. All assays were performed using this mixture of actin variants, allowing clear distinction between formin-elongated actin filaments in the presence of profilin, which appeared green due to the low affinity of the cysteine-labelled actin for profilin^62^ versus non-formin-associated/freely growing filaments, which appeared reddish to yellow due to lack of preference for any of the two coloured actin variants (see Fig. 1e). At the imaging conditions used, the ATTO565-labelled probe was brighter than the ATTO488-labelled one. Thus, in case of nucleation assays, red channel images displayed in black and white were shown for better visibility (Fig. 1b). Images from a Nikon Eclipse TI-E inverted microscope equipped with a TIRF Apo × 60 objective were captured every 4 s with exposure times of 40 ms using a Ixon3 897 EMCCD camera (Andor) for at least 10 min and up to 20 min. Time-lapse movies of at least two (10–100 nM FMNL fragments) and up to three (1 nM FMNL fragments) positions per experiment were acquired using a motorized microscope stage. The pixel size corresponded to 0.27 μm.

### Analysis of TIRF data

Elongation rates of filaments were measured by manual tracking of growing filament barbed ends using ImageJ software: total numbers of analysed filaments (*n*) were: 24 from three movies per condition in experiments without PFN; 24 in actin controls including 5 μM PFN; 30 in experiments with 5 μM PFN and 10 nM FMNL2 or FMNL3; at least 35 (including reddish control filaments) from four movies per condition in experiments with 5 μM PFN and 1 nM of either formin variant, 25 in the same experiment without formin.

The nucleation efficacies were obtained by counting and averaging the number of actin filaments in an area of 80 μm × 80 μm at 180 s from two positions of three independent experiments for each condition.

For examining polymerization processivity, four experiments for each condition were analysed. Since many filament segments with sizes of >19,000 subunits had no determinable end points, they were grouped into one fraction. The total number of measured filament segments was 88 for FMNL2-21P, 63 for FMNL3 and 101 for FMNL2-8P.

### RNA interference

RNAi in mammalian cells was done using vectors driving the expression of short hairpin (sh) RNAs. RNAi vectors were purchased from InvivoGen. Targeting sequence causing knockdown of FMNL2 was 5′-GGAAGTCTGCGGATGAGATAT-3′. Knockdown of FMNL3 was achieved using targeting sequence 5′- GGTGCAGATTCAAGCGTACCT-3′. Combined transfection of these vectors was used to generate double-knockdown cells, denoted as FMNL2+FMNL3 RNAi. Targeting sequence 5′-GGAATTAAGAAGGCGACAAGT-3′ was found to affect expression of both FMNL2 and FMNL3. Double-knockdown with the latter was denoted FMNL2/3 RNAi. All targeting sequences were fused into the psiRNA-h7SK vector backbone encoding a GFP or mCherry variant, respectively. A vector harbouring a scrambled sequence was used as RNAi control (psiRNA-h7SK control). For FMNL formin knockdown, B16 cells were co-transfected with respective psiRNA-vectors plus a plasmid conferring resistance to puromycin (pPUR, Clontech). Sixteen hours post-transfection, cells were trypsinized and subconfluently seeded using B16 medium supplemented with 2.5 μg ml^−1^ puromycin to eliminate non-transfected cells. Protein rundown was routinely documented by western blotting. Knockdown efficiency was found to be optimal 4 days after transfection of respective constructs, on average; so all analyses were performed at this time point.

### CRISPR/Cas9-mediated genome editing

Selected DNA target sequences (exon 1 in case of *FMNL2* and *FMNL3*) were pasted into a CRISPR design tool (http://tools.genome-engineering.org). Resulting potential target sites with a high efficiency score were used for designing the sgRNA constructs (20 nucleotides). In case of *FMNL3*, used target sequence was 5′-GCAAGACGCCGATGCCCGAGC-3′. Genome editing of *FMNL2* was performed using guides 5′-ATGCCCGAGCCAGGTGAACT-3′ (#2) or 5′-GATGCCCGAGCCAGGTGAAC-3′ (#5). Respective sequences were ligated into expression plasmid pSpCas9(BB)-2A-GFP (Addgene plasmid ID:48138) using BbsI (ref. 63). Sequence validation of CRISPR plasmids was performed using sequencing primer 5′-GCACCGACTCGGTGCCAC-3′.

For generation of genome-edited cell lines, B16 cells were transfected with mixtures of respective CRISPR plasmid and pPUR conferring puromycin resistance. Afterwards, transfected cells were selected with medium containing 2.5 μg ml^−1^ puromycin for 4 days. In order to generate single cell colonies, cells were extensively diluted and cultured in conditioned medium until they reached macroscopically visible size. Single colonies were picked and expanded. Cell lysates were generated and tested for FMNL expression by western blotting. Cell colonies that lacked expression of the target protein were genotyped as follows. Upon expansion and growth to confluence in 6 cm dishes, cells were trypsinized, pelleted and lysed adding 500 μl lysis buffer (100 mM Tris pH 8.5, 5 mM EDTA, 0.2% SDS, 200 mM NaCl) containing 2.5 μl proteinase K (20 mg ml^−1^). Samples were incubated overnight at 55 °C. Nucleic acid extraction was performed by a standard phenol/chloroform precipitation procedure. Isolated genomic DNA was used as template in PCR reactions using Phusion High-Fidelty Polymerase as recommended by the manufacturer (New England Biolabs). Primers used for amplification of respective target gene loci were: 5′-CATGGGCAACGCGGGGAGC-3′ (fwd); 5′-CGAGGTGCTGCTCCCGCCAG-3′ (rev) in case of *FMNL2* and 5′-CGATGGGCAACCTGGAGAGCACC-3′ (fwd); 5′-GGAATGGAATTCCGGCAGCGGACC-3′ (rev) in case of *FMNL3*. PCR products (∼350 bp) were examined on 2% agarose gels and appropriate samples purified with NucleoSpin Gel and PCR clean-up kit according to the manufacturer's instructions (Macherey&Nagel). DNA fragments were cloned into a zero blunt TOPO vector (Zero Blunt TOPO Cloning Kit for Sequencing, Invitrogen) as recommended by the manufacturer. Single bacterial colonies were inoculated overnight and plasmid DNA purified using NucleoSpin Plasmid kit (Macherey&Nagel). Sequencing of isolated plasmid DNA was carried out by MWG-Biotech (Ebersberg, Germany) using sequencing primer 5′-CAGGAAACAGCTATGAC-3′. Clones were examined for frameshift mutations and monoallelic or biallelic deletions/insertions. Mutations or deletions generating stop codons shortly downstream of the target site were defined as ‘null' alleles. Cell populations exclusively harbouring such alleles among >10 sequencing reactions were selected for further analyses. *FMNL2/3* double-KO B16 cells were generated by editing of *FMNL3* followed by *FMNL2*.

Genome editing in murine NIH 3T3 fibroblasts was performed with the same reagents and following the same procedures as with B16 cells, except that constructs targeting *FMNL2* and *FMNL3* were transfected simultaneously using jetPRIME reagent (Polyplus) according to the manufacturer's instructions, followed by selection of transfectants with 3 μg ml^−1^ puromycin. Isolated, individual clones were screened by western blotting, expanded and mutated loci sequenced as described above. Consistent with the established complex karyotype of NIH 3T3, with the majority of the genome being at least tetraploid^64^, we found four distinct null alleles in case of *FMNL3* (19 and 21 sequencing reactions for clone 9 and 46, respectively). The genome of clone 9 harboured *FMNL2* as multiple null alleles except for one in-frame deletion (lacking 6 nucleotides) and one wild-type allele (out of 17 reactions in clone 9). In clone 46, we exclusively detected mutated *FMNL2* alleles, all representing null alleles except for one reaction revealing an allele harbouring a 6 base pair and another revealing a 30 base pair in-frame deletion.

### Protein measurements and western blotting

For preparation of detergent-soluble extracts, cells were cultured to confluence, washed three times with ice-cold 1 × PBS (Gibco), lysed in ice cold lysis-buffer (140 mM KCl, 50 mM Tris-HCl (pH 7.4), 50 mM NaF, 10 mM Na_4_P_2_O_7_, 2 mM MgCl_2_, 1% Triton-X100, supplemented with one mini complete protease inhibitor pill (Roche)), and collected using a cell scraper. Finally, cell debris was removed by centrifugation at 20,000*g* for 15 min. Soluble protein concentrations were measured using standard Bradford assay and determined using a microplate reader (Varioskan Flash, Thermo Scientific). Western blotting was done according to standard procedures and using primary antibodies as follows: FMNL2/3 (Abcam; #ab57963; 1:1,000 dilution), GAPDH (Calbiochem; clone 6C5; #CB1001; 1:10,000 dilution), α-Tubulin (Synaptic Systems; clone 3A2; #302117; 1:50,000 dilution), phospho-cofilin (Cell Signaling; #3311; 1:1,000 dilution), cofilin 1 (KG60; kindly provided by Walter Witke; University of Bonn; Germany^65^; 1:500 dilution), FMNL1 (Abcam; #ab97456; 1:1,000 dilution). Secondary antibodies were purchased from Invitrogen. Primary antibodies in immunoblots were visualized with peroxidase-coupled anti-mouse IgG (Dianova; #115-035-062; 1:10,000 dilution) or anti-rabbit IgG (Dianova; #111-035-045; 1:10,000 dilution).

Uncropped images of most important western blots corresponding to Figs 2f and 4a are shown in Supplementary Fig. 19.

### Western blot intensity measurements

Detergent-soluble extracts from control or knockdown/knockout cells were analysed and compared according to their expression levels of cofilin 1 and phospho-cofilin 1 (inactive), respectively. Virtually identical results were obtained with total cell extracts (for representative example see Supplementary Fig. 14c). To quantify protein levels, intensity measurements on exposed western blot membranes were performed using MetaMorph software. Identical rectangular regions were drawn around protein bands of interest. To eliminate background signals contributing to the intensities measured, regions of the same size were measured in background areas and subtracted from individual, corresponding protein bands. Intensities of measured protein bands were normalized to those of corresponding loading controls (housekeeping genes, such as GAPDH). Corresponding data were plotted as bar charts using Excel 2010 (Microsoft).

### Immunofluorescence staining and quantification

For immunolabelling of Arp2/3 complex, B16 or NIH 3T3 cells were seeded onto glass coverslips coated with laminin (Sigma) or fibronectin (Roche), respectively. Laminin was diluted to 25 μg ml^−1^ in 50 mM Tris (pH 7.4), 150 mM NaCl and incubated for 60 min on coverslips before cell seeding. Fibronectin was diluted to 25 μg ml^−1^ in PBS. Cells were allowed to adhere overnight. Next, cells were fixed with prewarmed, 4% paraformaldehyde (PFA) in PBS for 20 min and permeabilized with 0.1% Triton-X100 in PBS for 1 min. Cells were blocked with 5% horse serum in 1% BSA in PBS, followed by staining with monoclonal p16A antibody^66^ (clone 323H3; undiluted hybridoma supernatant). Anti-FMNL2/3 staining shown in Supplementary Fig. 12 was performed using FMNL2/3-reactive antibody (Abcam; #ab57963; 1:50 dilution) after fixation of B16 as well as *FMNL2/3* KO cells treated with aluminium fluoride for 20 min, to stimulate lamellipodia^8^.

For fascin stainings using monoclonal antibody 55K2 (Santa Cruz Biotechnology; #SC-21743; 1:20 dilution), cells were fixed with ice-cold methanol. To visualize the actin cytoskeleton in these samples, an actin antibody (Sigma; #A2066; 1:50 dilution) recognizing its epitope upon methanol fixation was employed. For phalloidin stainings, cells were fixed with a mixture of 4% PFA and 0.25% glutaraldehyde for 20 min, enabling optimal preservation of the actin cytoskeleton. For Arp2/3 inhibition with CK666, B16 cells migrating on laminin o/n were transferred into fresh growth medium containing 210 μM inhibitor or vehicle control for 5 min, followed by fixation and phalloidin staining. Primary antibodies in immunofluorescence stainings were visualized with Alexa Fluor 488- (1:400 dilution) or Alexa Fluor 594-coupled (1:200 dilution) anti-mouse IgG (Invitrogen; #A11029 or #A11032, respectively). Secondary antibodies against rabbit primary antibodies were Alexa Fluor 488- (1:400 dilution) or Alexa Fluor 594-coupled (1:200 dilution) anti-rabbit IgG (Invitrogen, #A11034 or #A11037, respectively). Alexa Fluor 488- and Alexa Fluor 594-coupled phalloidin were also from Invitrogen (#A12379 and #A12381, respectively).

Fluorescence intensities of lamellipodial components such as actin or p16A were determined by defining a region restricted to the lamellipodium and a larger, extracellular region (defined as background). Average pixel intensities in background regions were subtracted from average intensities in lamellipodial regions. Net fluorescence intensities in lamellipodial regions were shown as raw data in box and whiskers plots.

Focal adhesions were visualized using a vinculin-reactive antibody (Sigma, #V9131, 1:250 dilution). To reduce unspecific background staining, cells were permeabilized with 0.3% Triton-X100 in 4% PFA/PBS for 1 min before 20 min fixation with 4% PFA/PBS. Quantifications of adhesion numbers and sizes were performed using ImageJ (particle analysis plugin) by manually setting appropriate thresholds. Owing to strong, nonspecific background fluorescence around the nucleus, only focal adhesions below lamellipodium and lamella region covering a large part of the cell surface from behind the lamellipodium till close to the nucleus were included into quantifications (see respective regions in Supplementary Figs 15 and 17). Data were plotted as bar charts using Excel 2010.

### Time-lapse microscopy and quantification of protrusion rates

Live cell imaging was done with B16 cells seeded on laminin (Sigma)-coated glass coverslips (25 μg ml^−1^) or with *Rac1*^*fl/fl*^ and *Rac1*^*−/−*^ (clone 3) mouse embryonic fibroblasts plated on fibronectin (25 μg ml^−1^, Roche). Cells were observed in an open heating chamber (Warner Instruments, Reading, UK) with a heater controller (Model TC-324 B, SN 1176) at 37 °C. Cells were maintained in microscopy medium (F12 HAM HEPES-buffered medium, Sigma) including 10% FCS, 2 mM L-glutamine and 1% penicillin/streptomycin. Conventional video microscopy was performed on an inverted microscope (Axiovert 100TV, Zeiss) equipped with an HXP 120 lamp for epifluorescence illumination, a halogen lamp for phase-contrast imaging and a Coolsnap-HQ2 camera (Photometrics). The microscope also comprises a filter wheel (LUDL Electronic Products LTD, SN: 102691 and driver SN: 1029595) and electronic shutters driven by MetaMorph software (Molecular Devices). Live cell images were obtained with × 40/1.3NA, × 63/1.4NA or × 100/1.4NA Plan apochromatic oil objectives as well as with × 100/1.3NA Plan Neofluar oil objectives.

Lamellipodium protrusion was determined based on kymographs generated from time-lapse images as follows: lamellipodia of B16 cells were recorded over a time period of at least 10 min acquiring images every 5 s. Kymographs were generated using MetaMorph software by drawing lines from inside the cell and across the lamellipodium. Corresponding regions from each time point of a time-lapse series were pasted next to each other along the *x* axis. Protrusion rates were determined by measuring advancement of lamellipodia tips (*y* axis) over time (*x* axis).

### Determination of actin assembly rates in lamellipodia

FRAP experiments were performed using an inverted Axio Observer microscope equipped with an automated stage, a DG4 light source (Sutter Instrument) for epifluorescence illumination, a VIS-LED for phase-contrast optics and a Coolsnap-HQ2 camera (Photometrics) driven by VisiView software (Visitron Systems). EGFP-actin was bleached in selected regions within lamellipodia employing the 2D-VisiFRAP Realtime Scanner (Visitron Systems) using 40–50 mW output power of a 405 nm diode laser (Visitron Systems). Movies were acquired at a rate of 3 s per frame.

Actin assembly rate is defined as the sum of actin retrograde flow and protrusion of the corresponding lamellipodial tip for a given time period. In case of FRAP experiments using EGFP-actin, actin assembly rate (μm min^−1^) equals the distance of fluorescence recovered from the lamellipodium tip over time^25^. Measured values were averaged for *n* cells, analysed and displayed as box and whiskers plots (Sigma plot 12.0, Systat Software).

As alternative to FRAP and EGFP-actin overexpression, actin assembly rates were determined in lamellipodia of B16 cells expressing EGFP-lifeact, by simply measuring distances (turquoise lines in Fig. 5c and Supplementary Fig. 8C) travelled by fluorescence inhomogeneities (open red circles in Fig. 5c and Supplementary Fig. 8C) within the actin meshwork over time.

### Random migration assays and lamellipodial persistence

For random migration assays, B16 or NIH 3T3 cells were seeded subconfluently into laminin- or fibronectin-coated μ-slide eight-well glass bottom microscopy chambers (Ibidi GmbH, Martinsried, Germany), respectively. After 3 h, regular medium was exchanged for microscopy medium (see above) and the chamber mounted onto an inverted Axio observer equipped with 37 °C incubator and CO_2_ atmosphere. Phase-contrast movies were acquired on different randomly chosen positions with a × 10/0.15NA Plan Neofluar objective and a frame rate of 4 frames per hour. For analysis, cells were manually tracked using ImageJ. DiPer software^67^ was employed for determining mean square displacement of cells and their directionality.

For some migration assays with NIH 3T3 fibroblasts, PVP (Sigma) at different concentrations was used to increase medium viscosity. PVP powder was added into prewarmed microscopy medium to a final concentration of 1–4% and sterile filtered. Prewarmed PVP-containing microscopy medium was then used to replace regular medium immediately before start of movie acquisition.

Lamellipodial persistence (as in Supplementary Fig. 7) was determined using phase-contrast time-lapse microscopy of randomly migrating B16 cells, employing a × 25/0.8NA Plan Neofluar objective and a frame rate of 4 frames per minute. Lamellipodial persistence was expressed as time elapsed from initiation of the lamellipodium till its collapse.

### Atomic force microscopy (AFM) measurements

Cells were plated onto laminin-coated coverslips previously attached to petri dishes (Growth surface: 22.1 cm^2^, TPP Techno Plastic Products AG, Trasadingen, Switzerland) using silicone grease. Cantilevers (Type: PP-CONT (Pointprobe), NanoWorld, Neuchatel, Switzerland) were furnished with polystyrene beads (Radius: 6 μm, Polyscience Europe GmbH, Eppelheim, Germany) at their tips to achieve a defined contact shape. Beads were glued onto cantilever tips using M-Bond glue (M-Bond 610 Adhesive, Vishay Precision Group, Inc.) and a homemade microscope setup (Olympus Deutschland GmbH, Hamburg, Germany) equipped with two independently movable x–y–z stages. After baking at 85 °C overnight to ensure glue hardening, prepared cantilevers were mounted onto an inverted video microscope (Leica DM IRB, Leica Microsystems GmbH, Wetzlar, Germany) equipped with a commercial AFM scanning unit (Nano Wizard, JPK Instruments AG, Berlin, Germany). For calibration^68^, the vertical spring constant of the cantilever *k*_*c*_ was determined employing the thermal noise method, whereas the lateral spring constant 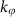 was calculated as follows: 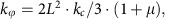
with *L* and *μ* denoting length and Poisson ratio of the cantilever, respectively (*μ*=0.25). Before measurements, calibrated cantilevers were placed in front of migrating cells and approached to the substrate with a pre-set force of 1 nN. Vertical and lateral voltage signals of cantilevers were recorded at a frequency of 100 Hz during measurements. The precise time point of contact between cell and cantilever was extracted from gathered force measurements, supported by phase contrast video microscopy using a frame rate of 0.5 Hz (objective: Leica × 40, Ph2, 0.55 NA; camera DFK 31AF03, The Image Source European GmbH, Bremen, Germany). The first peak after contact between cell and cantilever was defined as lamellipodial protrusion force and converted into force using respective calibration constants.

### Electron microscopy

For negative staining electron microscopy, cells were allowed to spread for 3–4 h on 2% Formvar-coated 200 mesh hexagonal gold grids (Agar Scientific). After spreading, the samples were immediately fixed and extracted with 0.25% glutaraldehyde and 0.5% Triton X-100 in cytoskeleton buffer (10 mM MES buffer, 150 mM NaCl, 5 mM EGTA, 5 mM glucose, 5 mM MgCl_2_, pH 6.8), then fixed for 5 min in 2% glutaraldehyde in cytoskeleton buffer and kept in 2% glutaraldehyde in cytoskeleton buffer at 4 °C until sample inspection. For electron tomography, grids were stained with 70 μl 4% sodium silicotungstate supplemented with 10 nm BSA-saturated colloidal gold. Tilt series of negatively stained cytoskeletons were acquired on a FEI Tecnai T20 microscope, operated at 200 kV. Automated acquisition of tilt series was driven by SerialEM version 3.4 with a typical tilt range from −60° to +60° using the Saxton tilt scheme based on 1° increments at a defocus value of −5 μm. Two tilt series around orthogonal axes were acquired for each tomogram and recorded on an Eagle 4k HS camera with a primary onscreen magnification of × 25,000. The resulting tilt series were processed using fiducials for alignment in IMOD software^69^. A custom-written Matlab-based software was used for automated filament tracking with threshold adjustment for nucleation sites as well as tracking steps according to a constant number of nucleation sites per lamellipodium area^70^. Four wild-type and six knock-out cells from three independent experiments were acquired and analysed using evenly spaced regions of 0.24 μm^2^ surface area.

### Data processing and statistical analyses

Brightness and contrast levels were adjusted using MetaMorph software (Molecular Devices Corp., Sunnyvale, CA, USA). Images were further processed for figure preparation using Adobe Photoshop CS4. Final figures were assembled with Photoshop CS4 or CorelDRAW Graphics Suite X7. Data analyses were carried out in ImageJ and MetaMorph, Excel 2010 (Microsoft) and Sigma plot 12.0 (Systat Software). Data sets were compared using the non-parametric Mann–Whitney rank sum test (Sigma plot 12.0). A probability of error of 5% (*p*<0.05; * in figure panels) was considered to indicate statistical significance. ** and *** indicated *p* values<0.01 and <0.001, respectively.

### Data availability

The authors declare that all relevant data supporting the findings of this study are available within the paper (and its supplementary information files). Any raw data can be obtained from the corresponding author (K.R.) on reasonable request.

## Additional information

**How to cite this article:** Kage, F. *et al*. FMNL formins boost lamellipodial force generation. *Nat. Commun.* **8,** 14832 doi: 10.1038/ncomms14832 (2017).

**Publisher's note:** Springer Nature remains neutral with regard to jurisdictional claims in published maps and institutional affiliations.

## Supplementary Material

Supplementary Information Supplementary Figures and Supplementary References

Supplementary Movie 1 FMNL3-EGFP is active and localizes to protruding lamellipodia.Related to Fig.1a. Time-lapse fluorescence and phase-contrast microscopy of B16-F1 cell transiently transfected with FMNL3-EGFP, which based on previous experiments with FMNL2 is expected to be myristoylated and thus fully regulated. Consistently, FMNL3-EGFP showed prominent accumulation at the tips of lamellipodia protruding at the cell front during migration. Time is given in minutes and seconds.

Supplementary Movie 2 Visualization of FMNL-mediated actin filament generation by TIRF microscopy.Related to Fig. 1b. Polymerization of 1 μM actin (10% ATTO488- and 5% ATTO565-labelled), visualized by TIRF-M alone or upon addition of 10 or 100 nM FMNL2-21P or FMNL3, as indicated. Note that filaments capped by formins grow considerably slower than control filaments, as known for FMNL2. Panels show areas of 80 × 80 μm. Time is in minutes and seconds.

Supplementary Movie 3 Analysis of formin-mediated filament elongation in the presence of profilin.Related to Fig. 1e. Polymerization of 1 μM actin (10% ATTO488- and 5% ATTO565-labelled) in the presence of 5 μM profilin (PFN) and 1 nM of formin fragments as indicated and visualized by TIRF1-M. Top left panel shows growth of control filaments in presence of 5 μM PFN. Filament segments elongated by FMNL2-8P (top right panel), FMNL2-21P (lower left panel) and FMNL3 (lower right panel) are growing in green and are marked by green arrowheads. Filament segments growing in red and thus with the rate of control filaments are marked by red arrowheads. Panels show an area of 80 × 80 μm each. Time is in minutes and seconds.

Supplementary Movie 4 Comparison of lamellipodium protrusion in control and FMNL2/3 knockdown cells.Related to Fig. 2. Phase contrast time-lapse microscopy performed on representative examples of mock RNAi- and FMNL2+3 RNAi-treated B16-F1 cells. Knockdown of FMNL2 and -3 (right panel) reduces average rate of lamellipodium protrusion. In addition, protrusion of lamellipodia in FMNL2+3 knockdown cells appears less continuous and more irregular than routinely observed in mock RNAitreated cells (see also Movie S6). Time is in minutes and seconds.

Supplementary Movie 5 Actin network assembly rates in FMNL2/3 knockdown versus control cells as determined by FRAP.Related to Supplementary Fig. 8a: Mock RNAi- or FMNL2/3 RNAi-treated cells transiently expressing EGFP-actin were subjected to fluorescence recovery after photobleaching (FRAP) experiments. Due to exclusive incorporation of actin fluorescence from the front, network assembly could be simply determined by summing up distances of network flow and protrusion for a given time period shortly after the bleach. Surprisingly, actin assembly rates of FMNL2/3 knockdown cells were indistinguishable from controls. Note, however, that full recovery of fluorescence in the lamellipodium of FMNL2/3 knockdown cells was reached even earlier than in controls, simply due to the fact that FMNL2/3 knockdown cells exhibited narrower lamellipodia on average than control cells (see Figs. 3a, b). Time is in minutes and seconds.

Supplementary Movie 6 FMNL2/3 knockdown cells exhibit irregular, fluctuating protrusion of lamellipodia.Related to Supplementary Fig. 6c, d: Representative example of time-lapse fluorescence microscopy of lamellipodia formed by mock versus FMNL2/3 RNAi-treated cells expressing EGFP-lifeact. Note that the FMNL2/3 knockdown cell exhibits a narrowed, less efficiently protruding and fluctuating lamellipodium. However, actin assembly rate is unchanged compared to controls. Bar equals 3 μm and time is given in minutes and seconds.
