# Supplementary material for: FMNL formins boost lamellipodial force generation
Source: Nat Commun. 2017 Mar 22;8:14832. doi: 10.1038/ncomms14832 (PMC5364437; doi:10.1038/ncomms14832)
Supplement: Supplementary Information — Supplementary Figures and Supplementary References [file ncomms14832-s1.pdf]

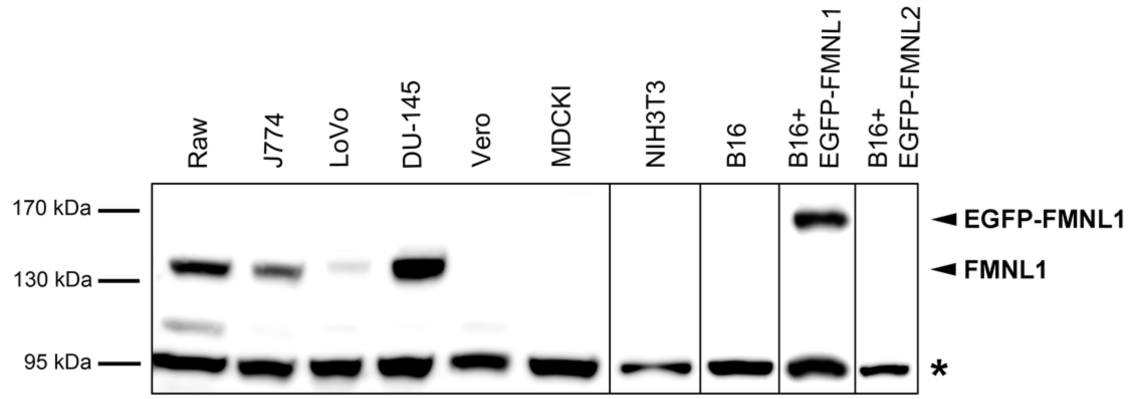

Supplementary Fig. 1. The FMNL subfamily member FMNL1 is not expressed in B16-F1 cells. Western Blotting of cell extracts as indicated using anti-FMNL1 antibody. FMNL1 is prominently expressed in cell lines of hematopoietic origin (Raw; J774), and to a variable extent in carcinogenic cell lines of epithelial origin (e.g. DU-145; Lovo), as expected[1, 2, 3], but is not detectable in B16-F1 melanoma cells or NIH 3T3 fibroblasts. Co-transfection with EGFP-tagged FMNL1 confirmed specific interaction with this FMNL subfamily member, but not with FMNL2. The cross-reaction at app. 95kDa (asterisk) was used as loading control.

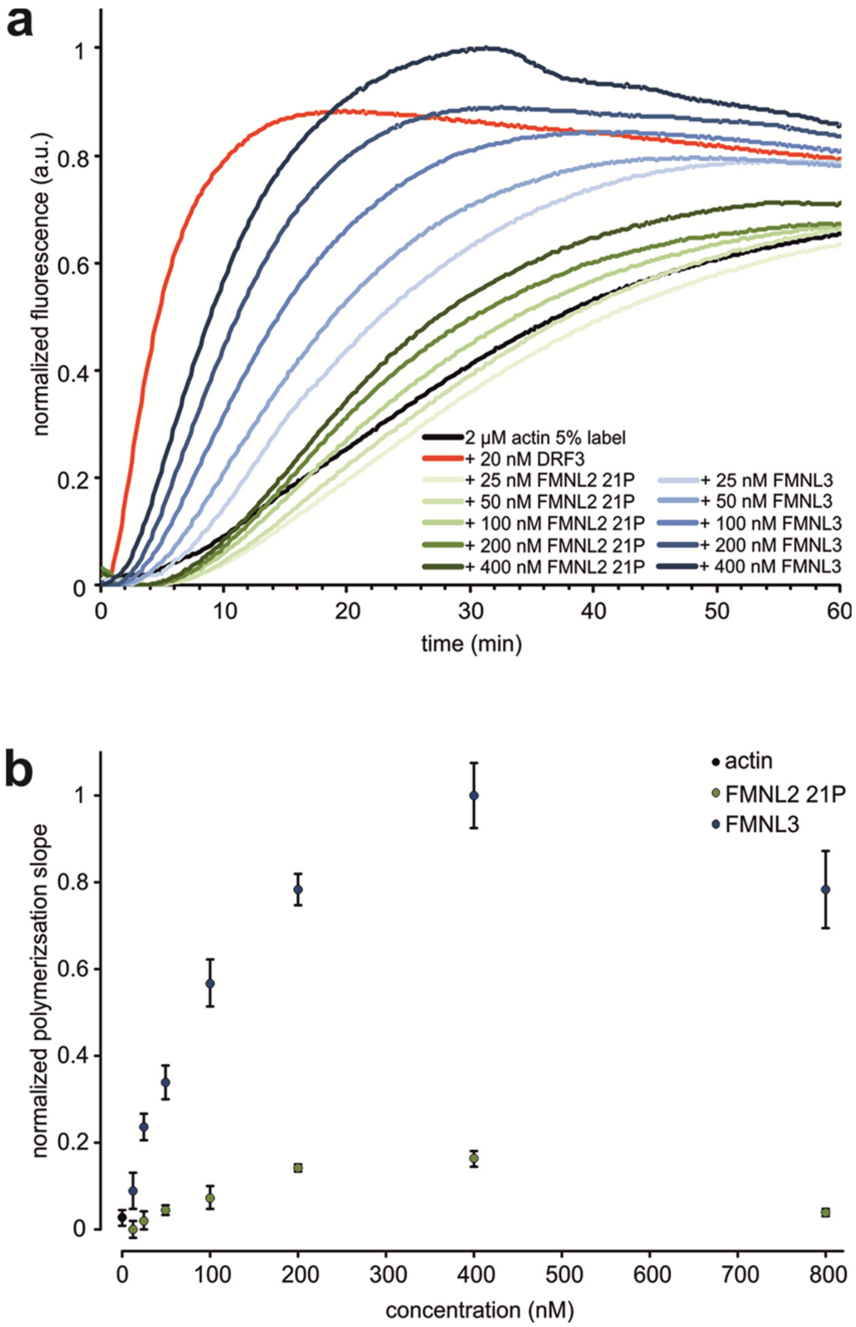

Supplementary Fig. 2. Comparison of FMNL2 *versus* FMNL3 in pyrene-actin assembly assays. (**a**) Polymerization of pyrene-actin in absence of profilin (PFN). 2 μM G-actin (5% pyrene-labelled) was polymerized in 1x KMEI buffer in presence of various formin fragments with concentrations as indicated. FMNL3 increases pyrene-actin polymerization in a concentration-dependent manner, whereas FMNL2-21P slightly suppresses actin polymerization at lower concentrations and stimulates actin polymerization weakly at higher concentrations. For both formins, polymerization activity diminishes at very high concentrations [800 nM], presumably due to sequestration of actin monomers by an excess of formin dimers. The C-terminus of Drf3 [20nM] is used as positive control for a formin with high polymerization activity. (**b**) Normalized slopes of polymerization curves exemplarily shown in (a) and plotted against the concentration of the respective formin fragment, as indicated. n = 4, error bars indicate SD.

26

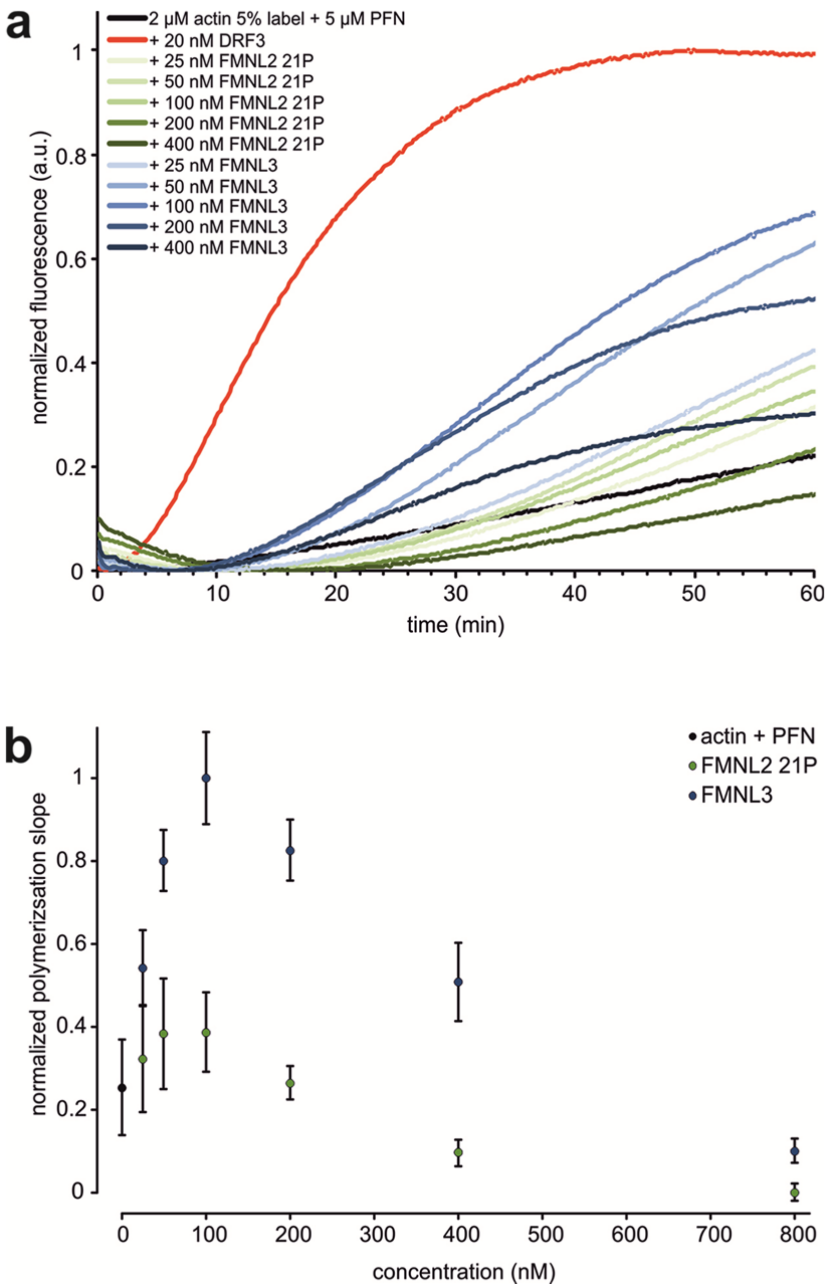

Supplementary Fig. 3. Comparison of FMNL2 *versus* FMNL3 in pyrene-actin assembly assays in presence of profilin. (**a**) Polymerization of pyrene-actin in the presence of PFN. 2 µM G-actin (5% pyrene-labelled) was polymerized in 1x KMEI buffer in presence of 5 µM PFN and various formin fragments with concentrations as indicated. FMNL3 and FMNL2-21P stimulate pyrene-actin polymerization in a concentration-dependent manner even in the presence of profilin, except for formin fragment concentrations ≥200nM, which show decreased fluorescence possibly due to selective polymerization of non-tagged profilin-actin complexes. The C-terminus of Drf3 [20nM] is used as positive control for a formin variant with high polymerization activity. (**b**) Normalized slopes of polymerization curves exemplarily shown in (a) and plotted against the concentration of the respective formin fragment as indicated. n = 4, error bars indicate SD.

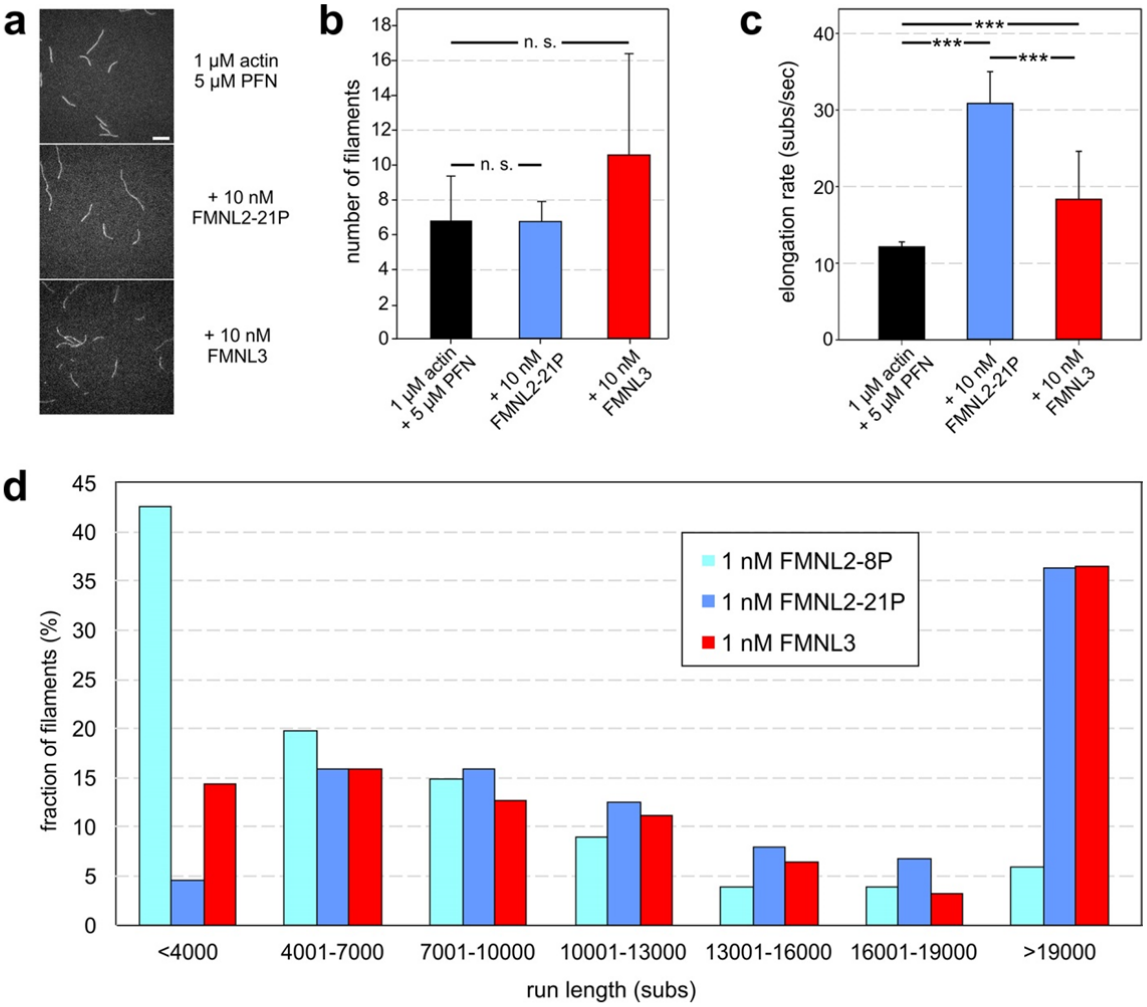

Supplementary Fig. 4. Additional TIRF-M experiments on FMNL activity in presence of profilin. (**a**) Time-lapse micrographs show representative areas analyzed in (b). 1 µM G-actin (10% labelled with ATTO488, 5% labelled with ATTO565) was polymerized with 5 µM profilin in the absence and presence of FMNL2-21P or FMNL3 with concentrations as indicated. Red channel displayed in greyscale; time, min; bar, 10 µm. (**b**) Nucleation activities of FMNL2-21P and FMNL3 in the presence of profilin. Profilin suppressed average nucleation of actin filaments in the absence of formins, but also dampened formin-induced nucleation significantly. Filament numbers were counted 3 min after G-actin addition. Error bars represent SD. (**c**) Average elongation rates of actin filaments in the presence of 5 µM PFN and FMNL2-21P or FMNL3 at 10 nM compared to control filaments. Both formins significantly enhance barbed end elongation in the presence of profilin-actin, with FMNL2-21P being significantly more efficient than FMNL3. Error bars represent SD. (**d**) Relative distributions of filament segment lengths elongated by either formin fragment in experiments like the one shown in Fig. 1e. As filament segment lengths directly depend on formin processivity, the data reveal FMNL2-8P to be substantially less processive than FMNL2-21P and FMNL3.

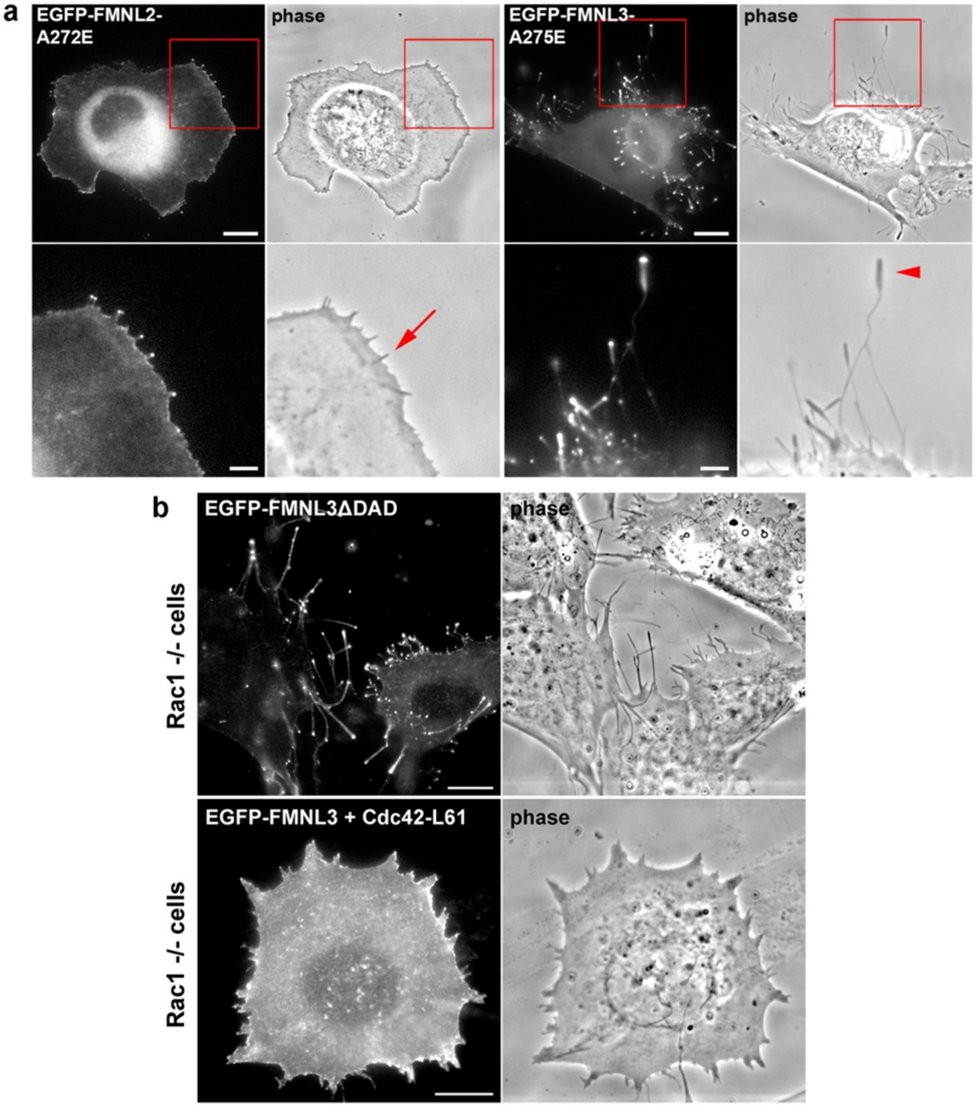

Supplementary Fig. 5. Active FMNL3 can induce club-shaped filopodia, but is unable to restore lamellipodia in Rac1-deficient cells. (**a**) Fluorescence and phase contrast overview images of B16-F1 cells expressing EGFP-tagged, constitutively active variants of FMNL2 (A272E) and FMNL3 (A275E), as indicated (top panels). Red rectangles mark insets magnified in bottom panels. Both formins are enriched at the tips of filopodia. However, as opposed to FMNL2 that just localizes to canonically shaped filopodia present in physiological numbers and co-existing with lamellipodia (red arrow), active FMNL3 generates excess numbers of club-shaped filopodia -frequently at the expense of lamellipodia - that can grow unusually long (red arrowhead). Formation of club-shaped filopodia has previously been linked to actin nucleation *in vivo*, in the context of activated Drf3/mDia2[4, 5], and would fit the increased nucleation capacity of FMNL3 as compared to FMNL2 observed *in vitro* (Fig. 1). Bars in overview panels are 10 µm, and in magnified insets 3 µm. (**b**) Fluorescence- and phase-contrast images of *Rac1*$^{-/-}$ cells deficient in lamellipodia formation[6], and transfected either with EGFP-FMNL3ΔDAD (top panel), or co-transfected with wild type EGFP-FMNL3 and constitutively active, myc-tagged Cdc42 (Cdc42-L61). Note that neither of the aforementioned treatments can restore the formation of lamellipodia-like structures in these cells, in spite of the prominent accumulation of the formin at the cell periphery, in particular upon co-overexpression of EGFP-FMNL3 and Cdc42. Active FMNL3ΔDAD mainly drives the formation of club-shaped filopodia. Bars represent 10 µm.

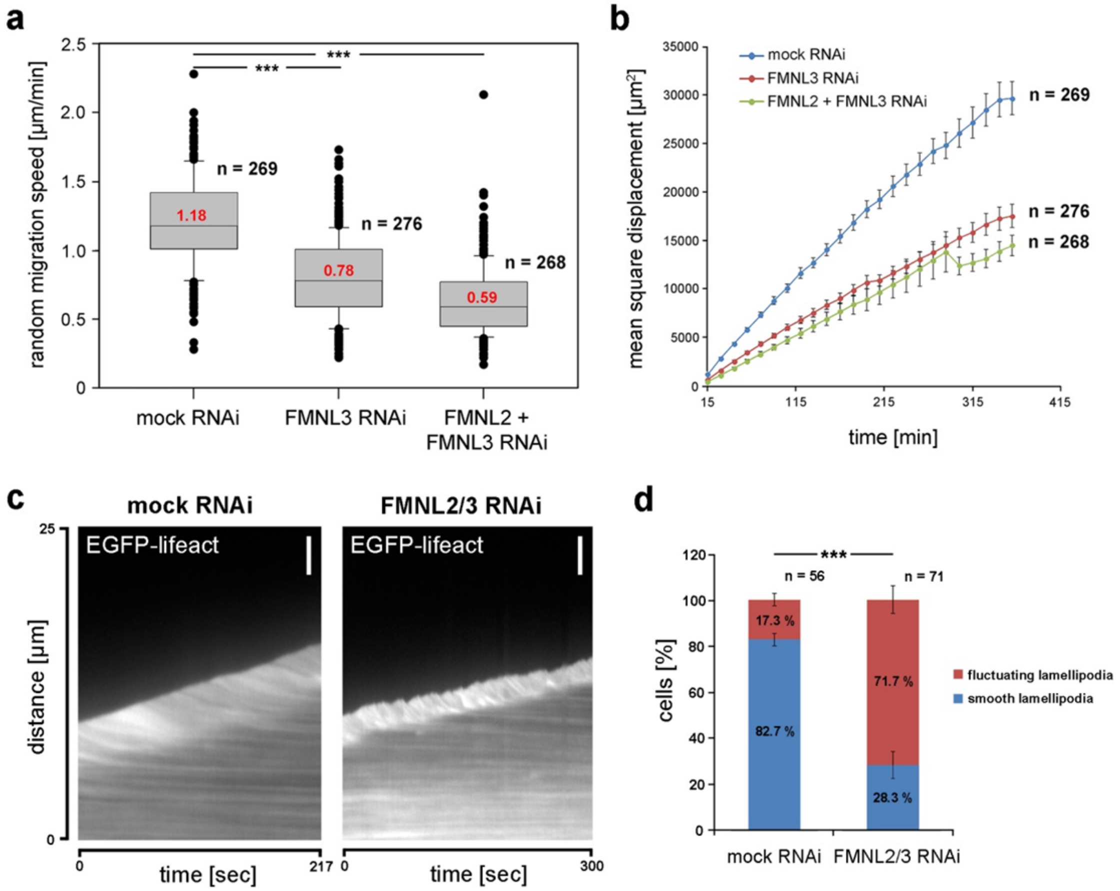

Supplementary Fig. 6. FMNL2/3 knockdown cells display reduced migration and a higher frequency of cells with irregular, fluctuating lamellipodia protrusions. (**a**) Box and whiskers plots summarizing quantitation of random migration rates (µm min -1) of FMNL3 alone or FMNL2+FMNL3 double-knockdown cells as compared to controls. FMNL3 knockdown reduces migration of B16-F1 cells, but the effect is enhanced further upon knockdown of both FMNL2 and FMNL3. (**b**) Mean square displacement (µm^2) over elapsed time (min) plotted on a linear scale. n gives the number of trajectories corresponding to mock RNAi- (blue), FMNL3 RNAi- (red) and FMNL2+3 RNAi- (green) treated cells, respectively. Note that average surface areas explored by control cells over time are much larger than those explored by FMNL knockdown cells. Error bars for each time point are standard errors of means. (**c**) Kymographs depicting forward advancement of the lamellipodium over time of B16-F1 cells RNAi-treated as indicated, and co-transfected with EGFP-lifeact used for time-lapse imaging of the F-actin cytoskeleton. Note the strongly fluctuating lamellipodial edge of the FMNL2/3 knockdown cell, greatly distinct from the smoothly protruding control cell. Scale bars correspond to 3 µm. (**d**) Bar diagram demonstrating percentage of cells harbouring smooth lamellipodial protrusions in blue *versus* fluctuating lamellipodial protrusions in red. Data were collected from three individual experiments (n gives the number of cells analyzed) and are shown as means ± standard errors of means. The difference in percentage of cells with fluctuating lamellipodial protrusions was confirmed to be statistically significant.

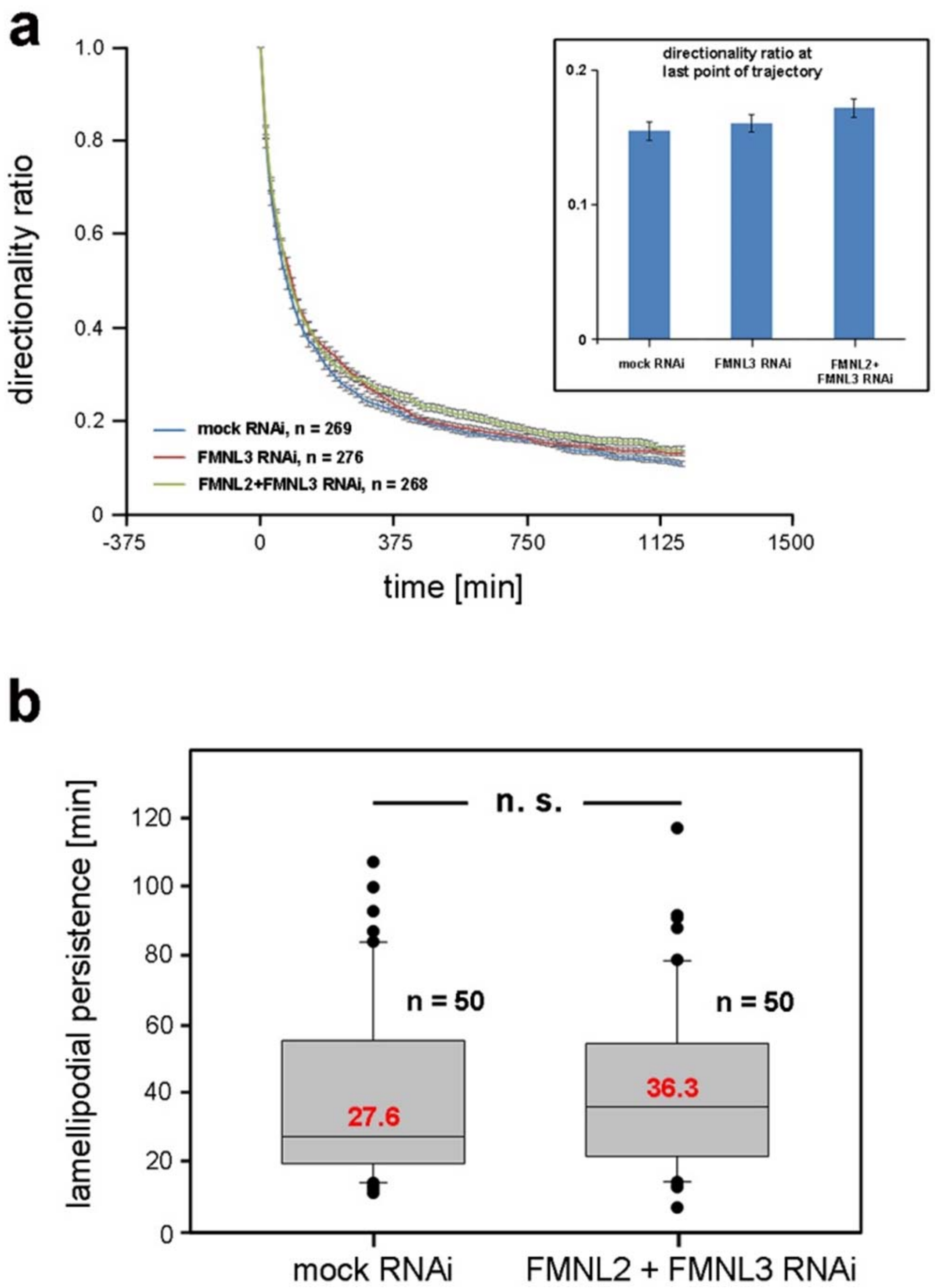

Supplementary Fig. 7. FMNL2/3 knockdown does not affect directionality of migration and lamellipodial persistence. (a) Graph shows directionality ratio over elapsed time [min]. In this case directionality is measured as the ratio of displacement to trajectory length. Note, there are no differences in directionality ratios of mock RNAi- (blue line), FMNL3 RNAi- (red line) and FMNL2+3 RNAi- (green line) treated cells as all curves decay rapidly in a similar fashion. Error bars are ±sem. (**b**) Lamellipodial persistence [min] of mock and FMNL2+3 RNAi-treated cells is shown in box and whiskers plots. Lamellipodial persistence was defined as the time of appearance to collapse of a given lamellipodium. N represents the number of analyzed lamellipodia and red numbers reflect median values. Datasets were confirmed not to be statistically different (n.s. = $p \geq 0.05$).

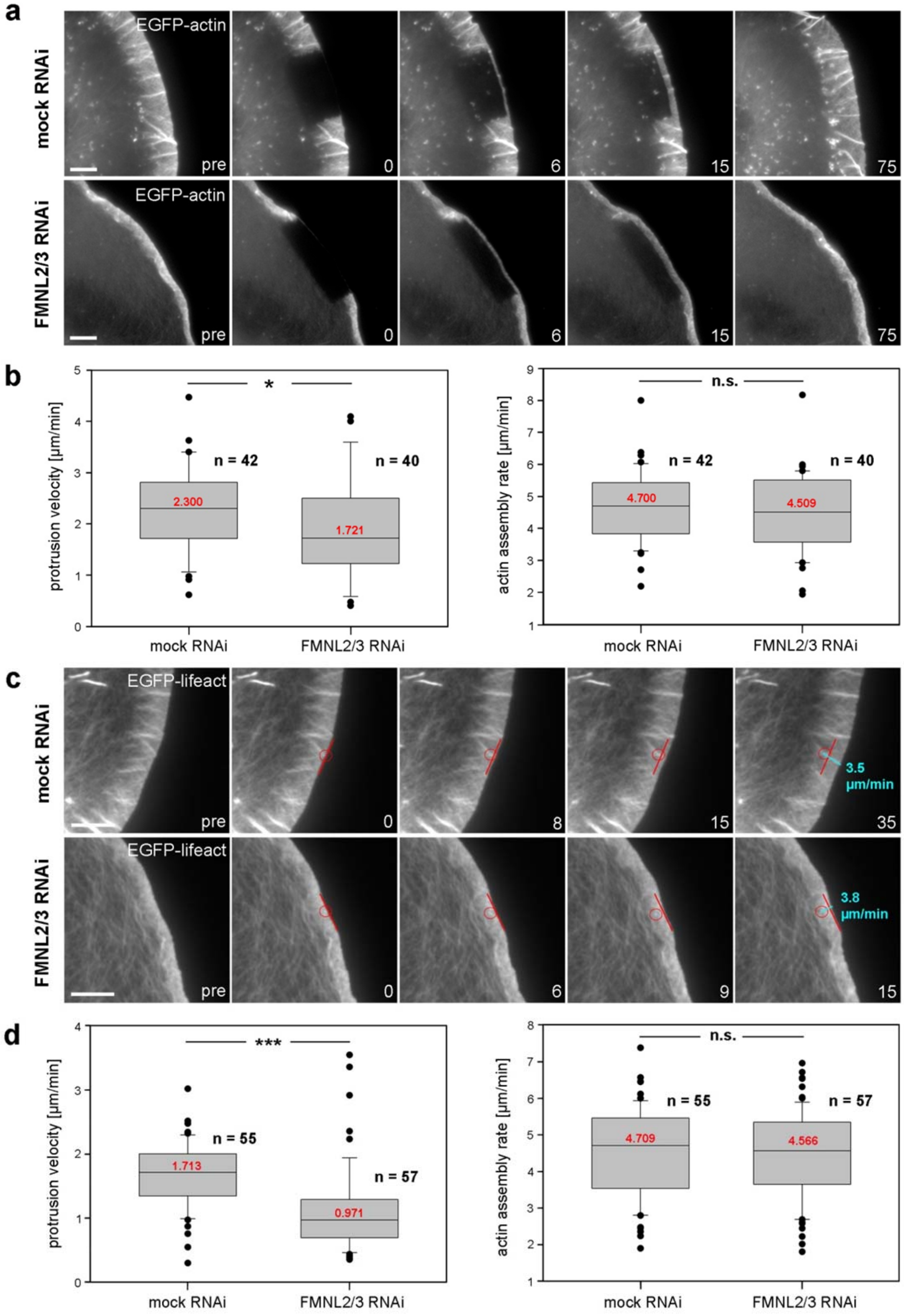

Supplementary Fig. 8. Rates of actin network polymerization are not affected in FMNL2/3 knockdown cells.

(**a**) Representative experiment showing fluorescence recovery after photobleaching (FRAP) of EGFP-actin in the lamellipodium of a mock or FMNL2/3 RNAi-treated cell, as indicated. Actin assembly rates were determined as the sum of retrograde actin flow and simultaneous protrusion of the bleached lamellipodial region. Pre corresponds to a time point shortly before photobleaching, time is in seconds, scale bars equal 5 µm. (**b**) Average protrusion velocities (left panel) or actin assembly rates (right panel) in mock *versus* FMNL2/3 knockdown cells overexpressing EGFP-actin. Box and whiskers plots and statistics were as described in Fig. 2. FMNL2/3 knockdown reduces average protrusion, but not speed of lamellipodial actin assembly. Note that, although statistically significant (* = $p \leq 0.05$), reduction of lamellipodium protrusion in EGFP-actin expressing cells was not as prominent as observed with non-expressing cells (Fig. 2), and increased in both mock and FMNL2/3 RNAi cells as compared to cells not transfected with EGFP-actin. (**c**) Time-lapse frames of mock and FMNL2/3 RNAi-treated cells transfected with EGFP-lifeact. Images show sections of respective lamellipodia prior to and during tracking of fluorescence inhomogeneities performed to determine lamellipodial actin assembly rates. Pre corresponds to a time point prior to analysis, whereas 0 marks the appearance of an inhomogeneity in the actin meshwork (red circle) and thus starting point of measurement. Red line marks position of the lamellipodium at measurement start and turquoise line in the last frame the measured distance of network translocation over the time period of measurement, corresponding to total network assembly rate. Time is in seconds, bars equal 5 µm. (**d**) Average protrusion velocities (left panel) or actin assembly rates (right panel) in mock *versus* FMNL2/3 knockdown cells expressing EGFP-lifeact. Box and whiskers plots are as above. Note that the speed of lamellipodial actin assembly is not reduced in FMNL2/3 knockdown cells (right panel), although protrusion velocity rates are significantly decreased (left panel).

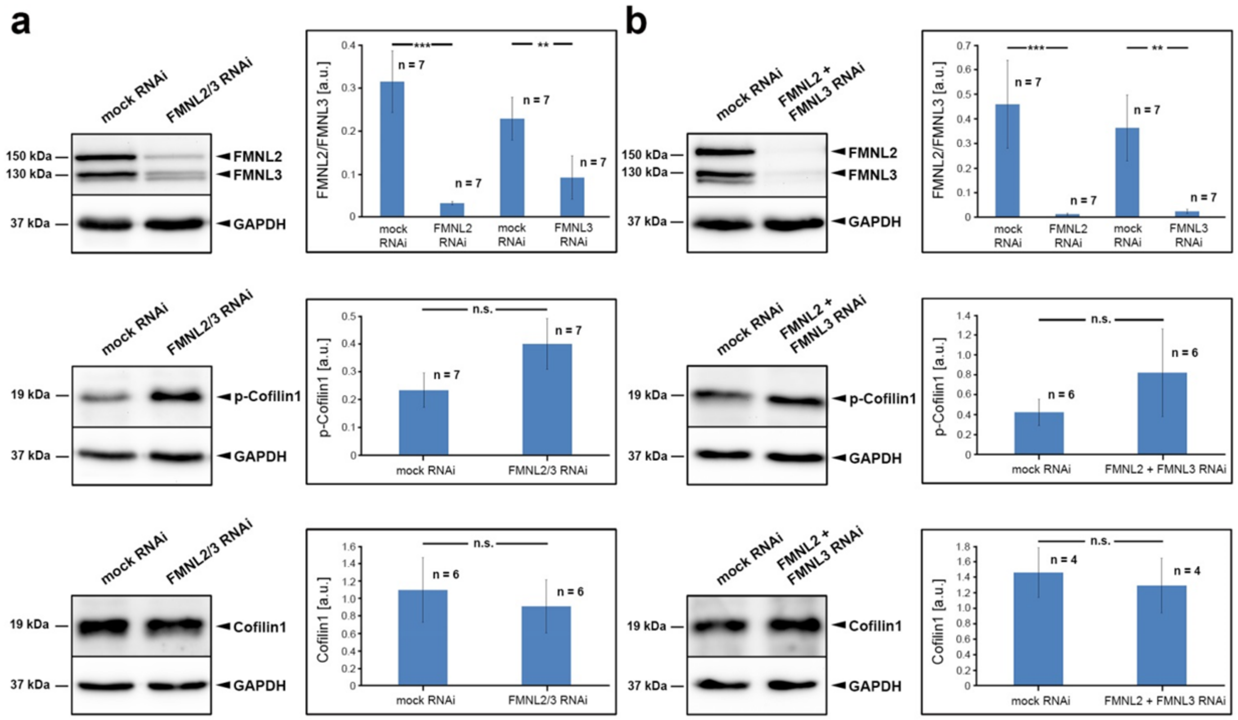

Supplementary Fig. 9. Reduced lamellipodium width of FMNL2/3 knockdown cells is not caused by increased cofilin activity. (**a**) Representative Western Blots and results from quantifications of FMNL2 and FMNL3 knockdown efficiencies relative to mock-plasmid transfected cells, as well as levels of phosphorylated, inactive cofilin1 and total cofilin1 in respective cell populations. Bar charts show arithmetic means of protein levels measured using ECL ChemoCam Imager HR 3.2 (INTAS Science Imaging Instruments GmbH, Göttingen, Germany), normalized to GAPDH. RNAi-mediated knockdown shown in (a) was achieved with one plasmid downregulating both FMNL2 and FMNL3 expression (see Materials and methods). (**b**) Identical experiments as in (a) with the exception that FMNL2 and -3 knockdown in each experiment was achieved by co-transfection of two plasmids each knocking down one of the two formins specifically. Note that knockdown of FMNL2 and -3 does not significantly change total cofilin levels, but appears to increase rather than decrease inactive cofilin levels. n denotes number of independently generated cell extracts, and error bars represent standard errors of means.

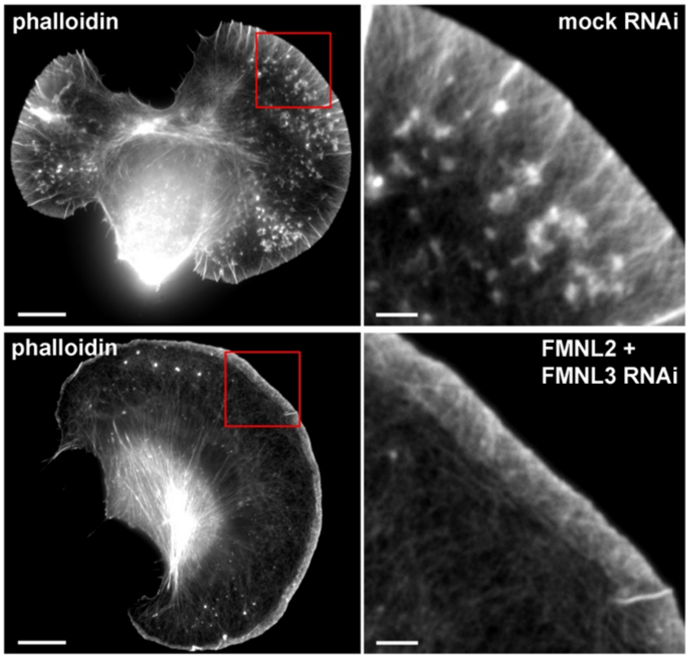

Supplementary Fig. 10. Combined knockdown of FMNL2 and -3 affects the structural organization of the lamellipodial network. Representative examples of actin organization in mock and FMNL2+FMNL3 RNAi-treated B16-F1 mouse melanoma cells upon fixation with 4% paraformaldehyde and 0.25% glutaraldehyde, followed by staining with fluorescent phalloidin. Red rectangles mark insets magnified and displayed on the right. The lamellipodial actin network of FMNL knockdown cells appears less well ordered and bundled than the network in control cells. This observation in RNAi-treated cells is comparable to the phenotype seen for FMNL2/3 CRISPR/Cas-KO cells (Fig 4b). Bars equal 10 µm in overview images and 3 µm in magnified insets.

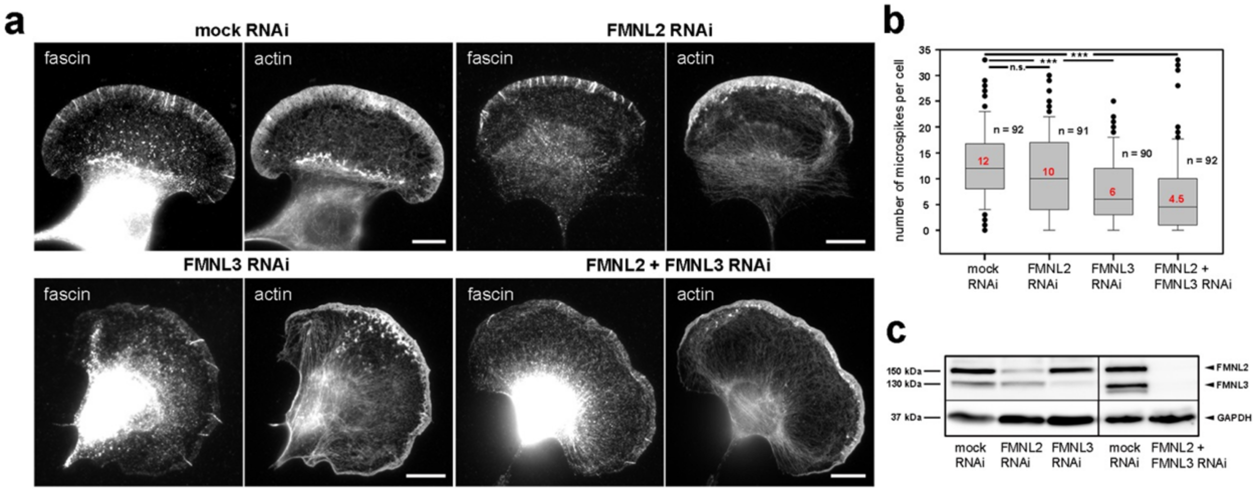

Supplementary Fig. 11. FMNL2/3 knockdown cells have reduced numbers of fascin-positive microspikes. (**a**) Immunolabellings of RNAi-treated B16-F1 cells using anti-fascin as well as anti-actin antibodies to visualize microspikes and the actin cytoskeleton, respectively. Scale bars equal 10 µm. (**b**) Quantitation of average microspike numbers from fascin images. (**c**) Representative, RNAi-induced reduction of protein expression as documented by Western Blotting from samples collected in parallel at the time point of experiments, using FMNL2/3-reactive antibody and GAPDH as loading control.

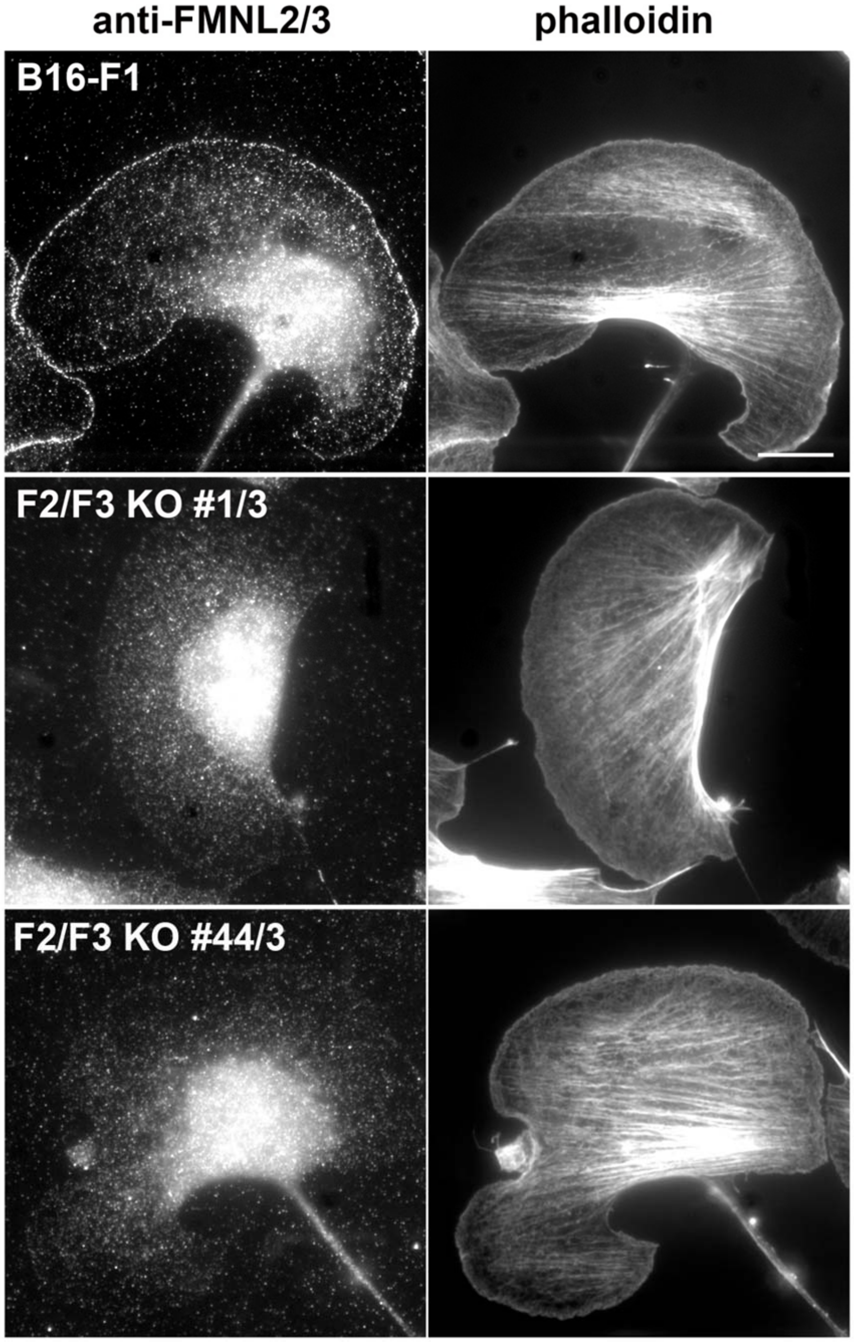

Supplementary Fig. 12. Immunolabelling of FMNL2 and -3 confirms their absence in double-KO B16-F1 cell lines generated by CRISPR/Cas9. B16-F1 control as well as *FMNL2/3* KO cells were stimulated with aluminum fluoride (see Methods), immunolabelled for endogenous FMNL2 and -3 and counterstained for the actin cytoskeleton by fluorescent phalloidin. Note that endogenous FMNL2 and -3 is enriched at the lamellipodium tip in case of B16-F1 wild type cells. Lamellipodial tip staining of FMNL2 and -3 is completely absent in both *FMNL2/3* KO cell clones. Bar is valid for all panels and equals 10 µm.

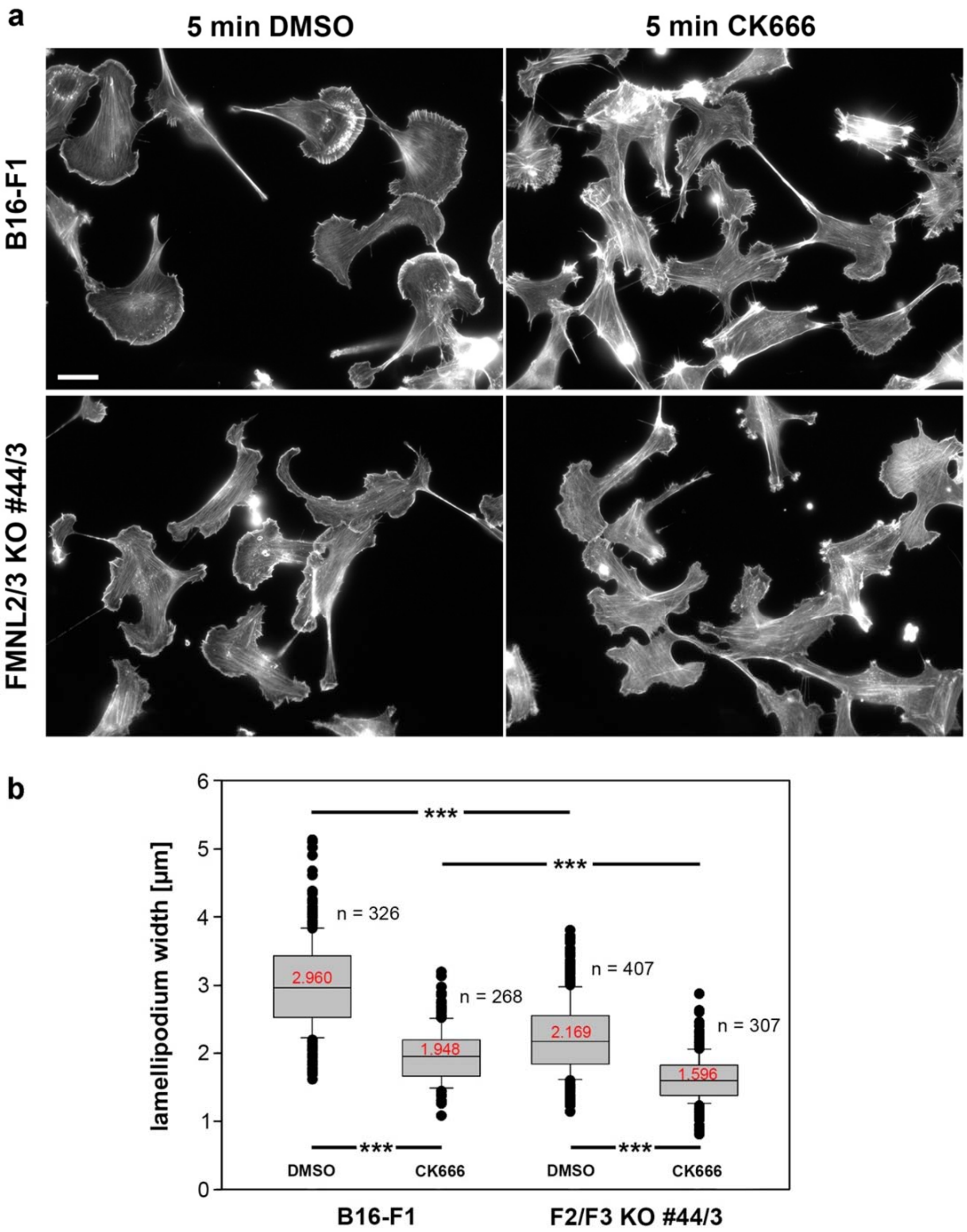

Supplementary Fig. 13. Arp2/3 complex inhibition reduces lamellipodium width in both control and *FMNL2/3* KO B16-F1 cells. (**a**) Representative images of B16-F1 wild type cells and *FMNL2/3* KO clone #44/3 treated for 5 min either with the Arp2/3 complex inhibitor CK666 (210 µM) or with DMSO, as indicated. After treatments, cells were fixed and stained for the actin cytoskeleton using phalloidin. Scale bar, valid for all panels, represents 20 µm. (**b**) Box and whiskers plot depicting lamellipodium width quantifications of cell populations illustrated in (a). Median values are given in red. n corresponds to the number of analyzed lamellipodia.

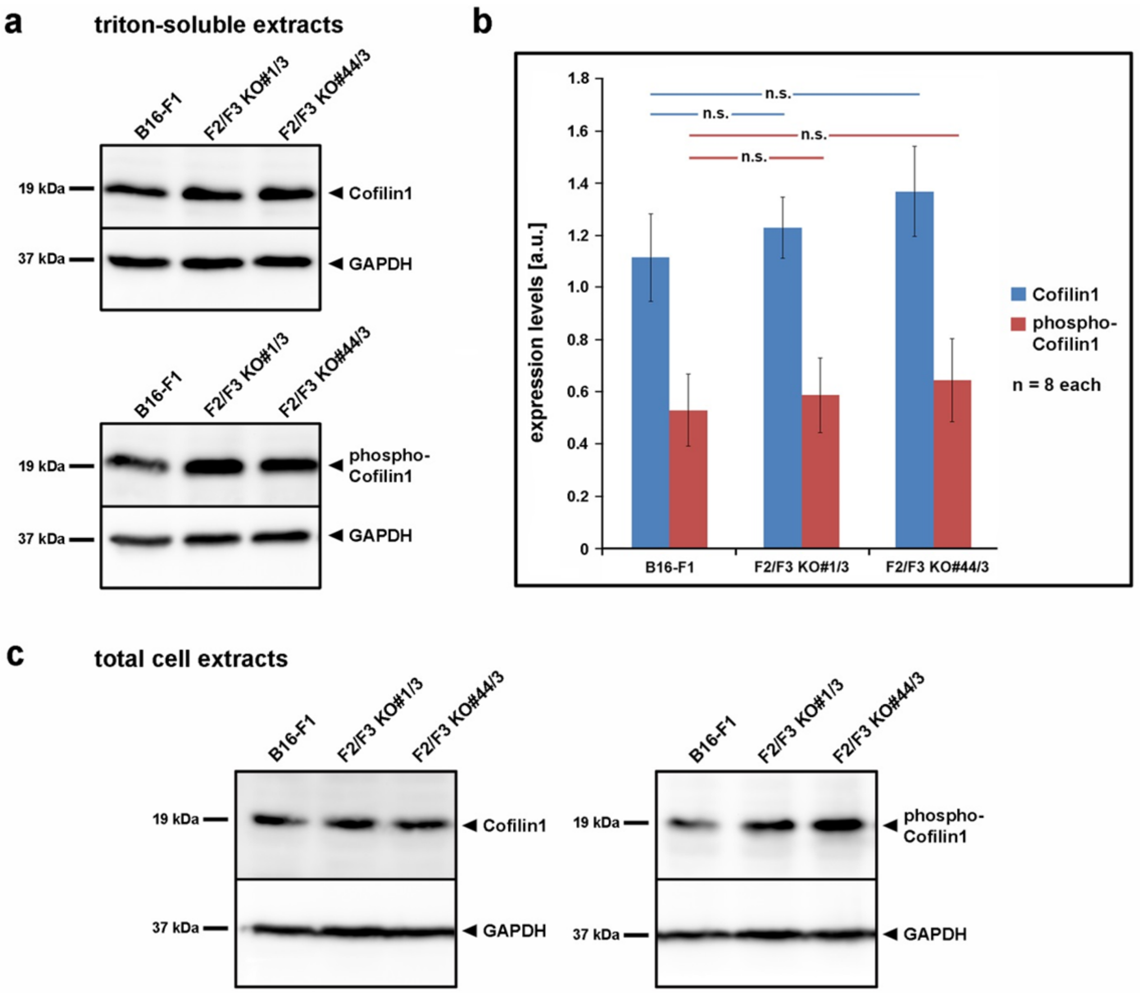

Supplementary Fig. 14. Lamellipodial narrowing in *FMNL2/3* KO B16-F1 cells is not due to increased levels of active cofilin. (**a**) Representative Western Blots showing levels of cofilin1 expression or of inactive cofilin due to phosphorylation at serine 3 (phospho-cofilin) in *FMNL2/3* double-KO cell lines as compared to B16-F1 controls in triton-soluble cell extracts. GAPDH was used as loading control. (**b**) Quantitation of Western Blot results done in analogy to data shown in Supplementary Fig. 9. Note that there are no significant changes in levels of cofilin1 or phospho-cofilin in KO cells compared to controls. (**c**) Western Blots performed in analogy to (a), with the exception that total cell extracts were used.

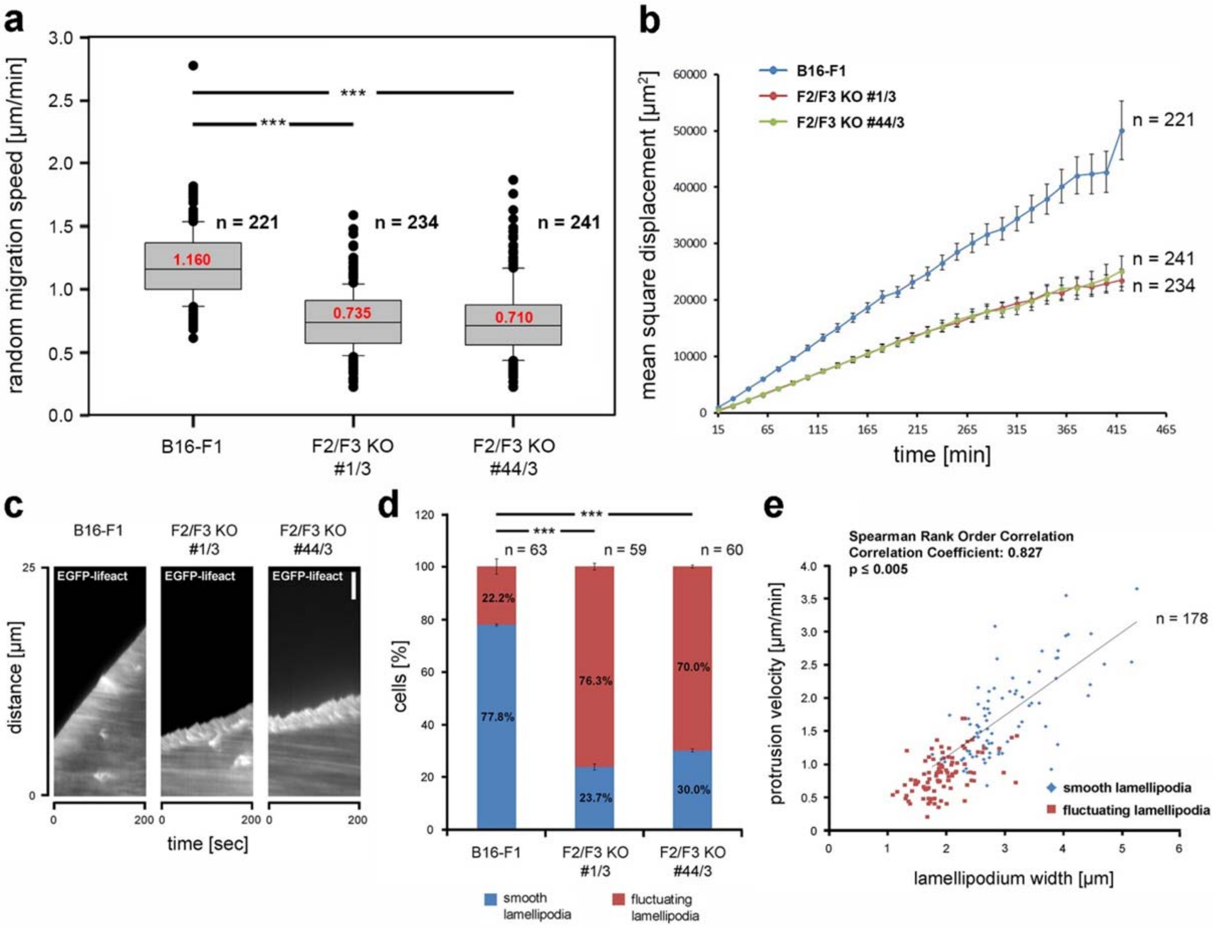

Supplementary Fig. 15. *FMNL2/3* double-KO B16-F1 cells display migration and protrusion defects. (**a**) Box and whiskers plots summarizing quantitation of random migration rates (µm min^{-1}) of double-knockout cell populations and wild type B16-F1 cells as indicated. (**b**) Mean square displacement ($µm^2$) over elapsed time (min) plotted on linear scale. n gives the number of trajectories corresponding to B16-F1 (blue), F2/F3 KO#1/3 (red), F2/F3 KO#44/3 (green), respectively. Error bars for each time point are standard errors of means. (**c**) Lamellipodial actin dynamics visualized by EGFP-lifeact expression combined with kymography. Displayed kymographs summarize protrusion of representative lamellipodia from KO cell lines and B16-F1 control cells as indicated. Scale bar equals 3 µm. (**d**) Quantitation of cells (%) harbouring smooth (blue) versus fluctuating (red) lamellipodia depicted in a bar diagram. Corresponding error bars represent standard errors of means. (**e**) Protrusion velocity plotted against lamellipodium width as derived from the kymography data shown in (c, d), combined with Spearman Rank Order Correlation analysis. Data are displayed irrespective of genotype and reveal a clear correlation between width of the lamellipodium and its protrusion speed ($p \leq 0.005$). Analyzed lamellipodia were additionally categorized either as smooth (blue rhombs) or fluctuating (red squares). These data illustrate that fluctuating lamellipodia are observed below specific threshold levels of protrusion rate and lamellipodium width.

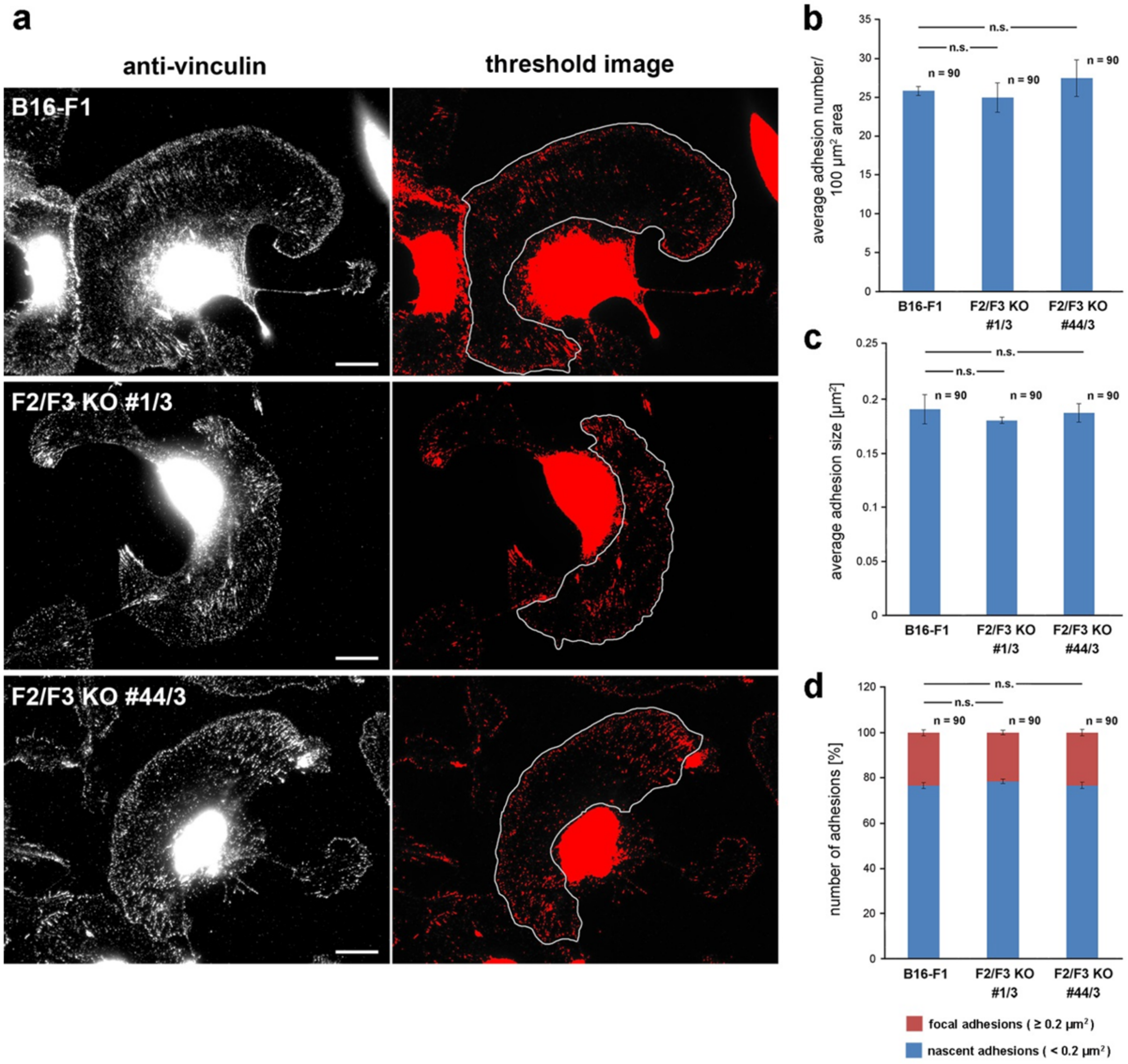

Supplementary Fig. 16. Loss of FMNL2 and -3 in B16-F1 cells does not affect focal adhesion number or size. (**a**) Immunolabelling of focal adhesions using anti-vinculin antibody (left panel) and corresponding thresholded images depicted on the right. Adhesions located in lamellipodial and lamellar regions (white outlines) were subjected to analysis shown in bar charts (b, c and d). Bars equal 10 µm. (**b**, **c**) Bar graphs illustrating average adhesion number per 100 µm^2 cell area (b) and average adhesion size (µm^2) (**c**), respectively. (**d**) Bar diagram showing relative distributions of nascent (< 0.2 µm^2, blue) *versus* focal adhesions (≥ 0.2 µm^2, red) in percent. Data were collected from three individual experiments (n gives the number of cells analyzed) and are shown as means ± standard errors of means.

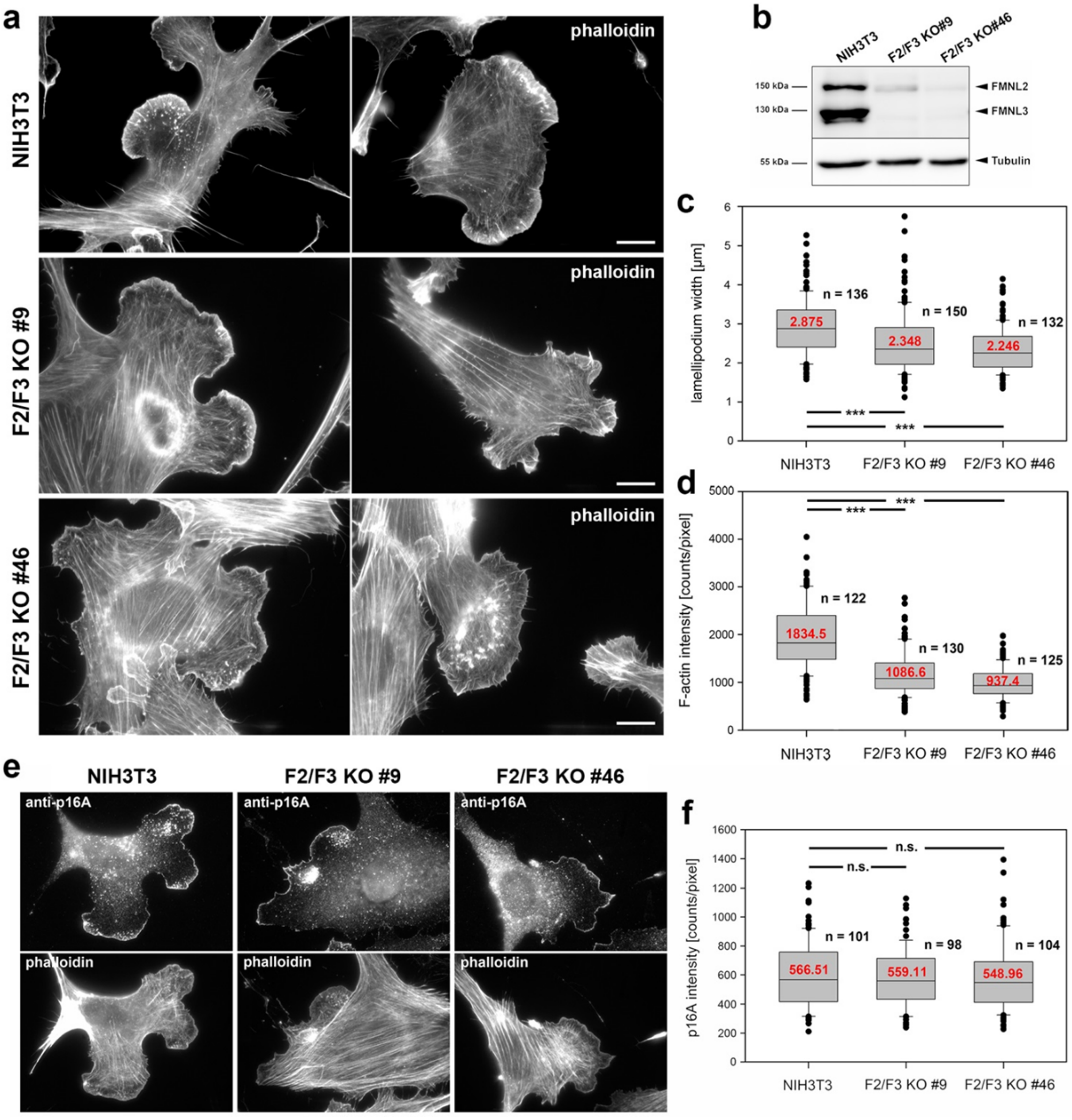

Supplementary Fig. 17. CRISPR/Cas9-treated NIH 3T3 fibroblasts display phenotypes analogous to B16-F1 *FMNL2/3* double-KO cells.

(**a**) Phalloidin stainings depicting two representative examples each of NIH 3T3 wild type and FMNL2/3 CRISPR/Cas-treated clones 9 and 46 (F2/F3 KO #9 and F2/F3 KO #46). Scale bars, 10 µm. (**b**) Western Blotting of cell extracts derived from NIH 3T3, F2/F3 KO clone #9 and #46, respectively. FMNL2/3-reactive antibody was used to validate CRISPR/Cas9-mediated FMNL2/3 depletion. Tubulin was used as loading control. (**c**, **d**) Average lamellipodium widths (µm) and F-actin intensities (photon counts pixel^{-1} after background subtraction) in denoted cell lines and summarized as box and whiskers plots, n, number of cells analyzed. (**e**) Immunolabelling of the Arp2/3 complex subunit p16A as indicated. (**f**) Quantitation of Arp2/3 complex intensities in lamellipodia of NIH 3T3 wild type and FMNL2/3 CRISPR/Cas clones, displayed analogous to F-actin in (d).

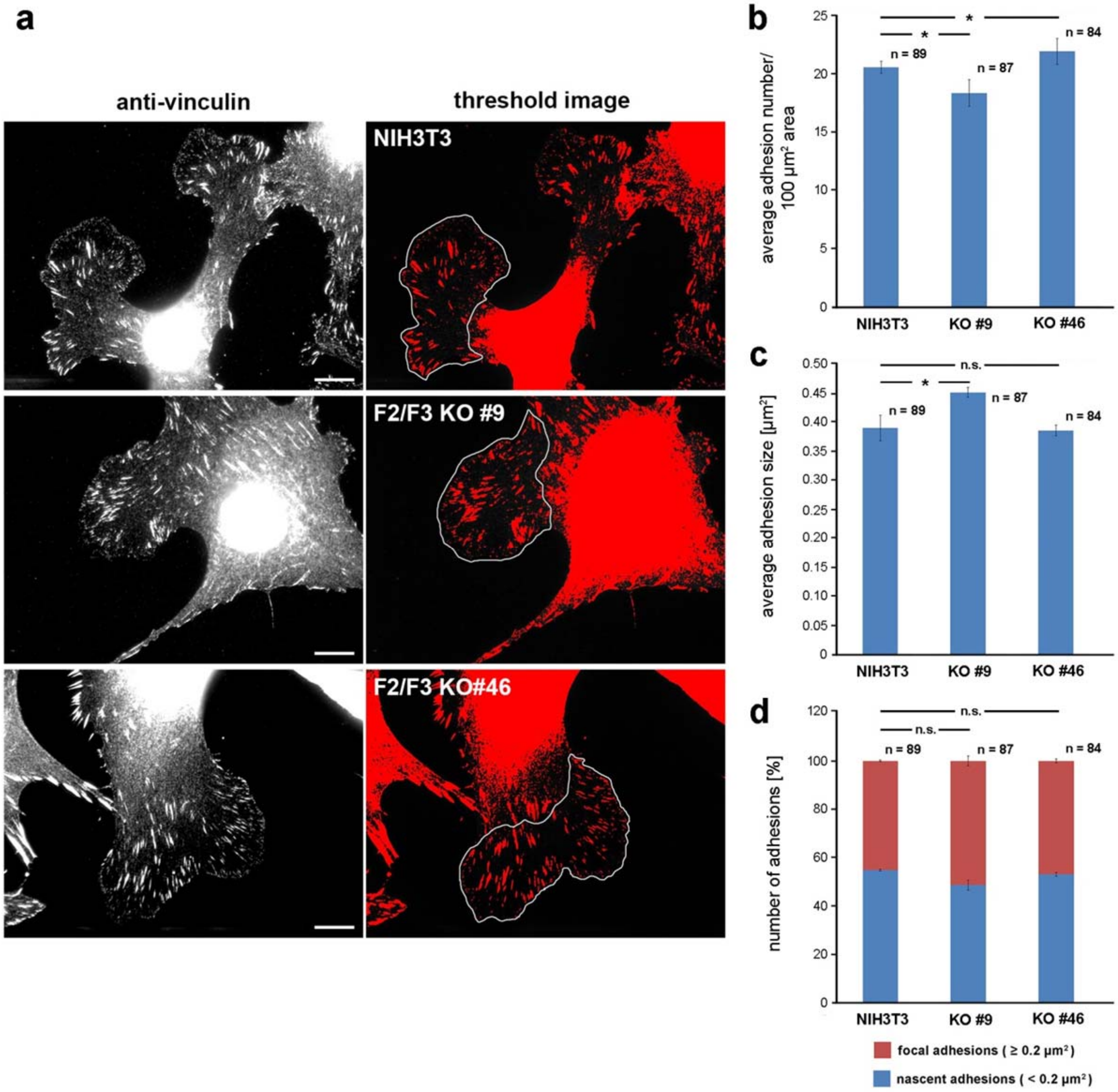

Supplementary Fig. 18. Depletion of FMNL2 and-3 in NIH 3T3 cells does not affect focal adhesion number or size. (**a**) Vinculin stainings of NIH 3T3 fibroblasts as indicated (left panel) and corresponding thresholded images (right panel). White outlines depict regions of focal adhesions in the lamellipodium and lamella that were used for quantifications shown in (b, c and d). Scale bars correspond to 10 µm. (**b**, **c**) Bar graphs illustrating average adhesion number per 100 μm^2 cell area and average adhesion size (μm^2), respectively, as indicated. (**d**) Bar diagram demonstrating relative distributions of nascent adhesions ($< 0.2\ \mu m^2$, blue) *versus* focal adhesions ($\geq 0.2\ \mu m^2$, red) in percent. Data were collected from three individual experiments (n gives the number of cells analyzed) and are shown as means ± standard errors of means.

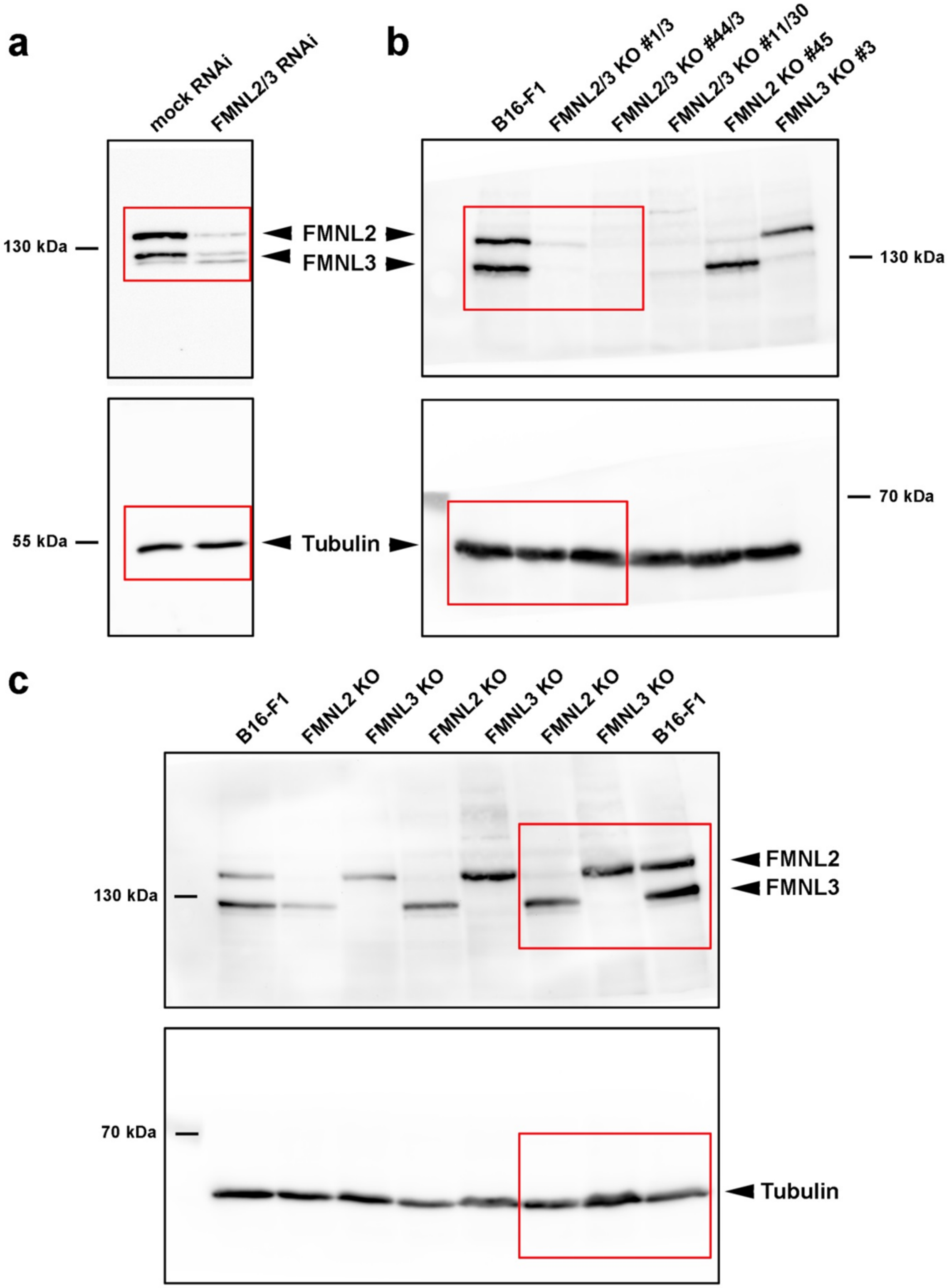

Supplementary Fig. 19. Uncropped images of important Western Blots. Representative, uncropped Western blot images of data shown in Fig. 2f (**a**) and Fig. 4a (**b**, **c**). Note that original gels were cut in half to transfer high and low molecular weight proteins separately due to distinct protein transfer times. Tubulin was used as loading control. Cropped regions are highlighted by red rectangles.

Supplementary references

1. Colon-Franco JM, Gomez TS, Billadeau DD. Dynamic remodeling of the actin cytoskeleton by FMNL1gamma is required for structural maintenance of the Golgi complex. *Journal of cell science* **124**, 3118-3126 (2011).

2. Kuhn S, Geyer M. Formins as effector proteins of Rho GTPases. *Small GTPases* **5**, e29513, doi: 10.4161/sgtp.29513 (2014).

3. Yayoshi-Yamamoto S, Taniuchi I, Watanabe T. FRL, a novel formin-related protein, binds to Rac and regulates cell motility and survival of macrophages. *Molecular and cellular biology* **20**, 6872-6881 (2000).

4. Block J*, et al.* Filopodia formation induced by active mDia2/Drf3. *Journal of microscopy* **231**, 506-517 (2008).

5. Yang C, Czech L, Gerboth S, Kojima S, Scita G, Svitkina T. Novel roles of formin mDia2 in lamellipodia and filopodia formation in motile cells. *PLoS biology* **5**, e317, doi: 10.1371/journal.pbio.0050317 (2007).

6. Steffen A*, et al.* Rac function is crucial for cell migration but is not required for spreading and focal adhesion formation. *Journal of cell science* **126**, 4572-4588 (2013).
